# Agronomic Performance and Elemental Uptake of a Potential Fertilizer Derived from Microwave-Treated Animal By-Products in *Lepidium sativum* and *Lactuca sativa*

**DOI:** 10.3390/plants15203120

**Published:** 2026-10-12

**Authors:** Alba Cao, Costantino Palmas, Giulia Urracci, Marco Dettori, Tiziana Pivetta

**Affiliations:** 1 Chemical and Geological Sciences Department, University of Cagliari, Cittadella Universitaria, 09042 Monserrato, CA, Italy; alba.cao@unica.it; 2 AGRIS–Caritas Protocol, Caritas Diocesana di Cagliari, Via Mons. Cogoni 9, 09121 Cagliari, Italy; palmascostantino@gmail.com; 3 Agenzia AGRIS Sardegna, Viale Trieste 111, 09123 Cagliari, Italy; gurracci@agrisricerca.it (G.U.); mdettori@agrisricerca.it (M.D.)

**Keywords:** animal by-products, organic fertilizer, PAPs, hydroponic culture

## Abstract

The aim of this study was to characterize a fertilizer derived from microwave-treated animal by-products consisting of processed animal proteins (PAPs), and to assess its preliminary agronomic performance. Germination tests, hydroponic cultivation, pot trials and elemental analyses of the fertilizer and plant tissues helped to identify suitable application rates, nutrient uptake response and potential limitations. The results reported in this study suggest that PAPs may affect plant growth and nutrient uptake in a species- and treatment-dependent way. In fact, among the tested concentrations, the most favorable responses were obtained with 0.75 g L^−1^ PAPs in hydroponic systems and 5 g kg^−1^ PAPs in pot trials, while higher concentrations progressively inhibited plant development. In the hydroponic experiments, *Lepidium sativum* treated with 0.75 g L^−1^ PAPs reached a mean length of 10 ± 1 cm, compared with 10.9 ± 0.4 cm for plants supplied with the commercial nutrient solution. In *Lactuca sativa* treated with 0.75 g L^−1^ PAPs, Ca and P concentrations were comparable to those measured in the plants treated with the commercial-nutrient-solution, whereas Mg increased by 60 % and K decreased by 73 %. The tested PAPs batch, deriving from fish-waste material, had an Hg content of 1.3 ± 0.1 mg kg^−1^ dry weight, highlighting the need for feedstock control and batch-specific compliance assessment. Despite the limitations of this preliminary study, the findings highlight promising application ranges, as well as critical methodological and compositional aspects to be addressed in future, in fully replicated investigations.

## 1. Introduction

The growing global demand for food, together with the increasing pressure on cultivable land, has intensified the need for more sustainable agricultural practices. To meet productivity demands, intensive farming systems are widely adopted, often involving the extensive use of chemical fertilizers [1] that, unfortunately, lead to important environmental issues such as water eutrophication, biodiversity loss, global warming, and stratospheric ozone depletion, as well as adverse effects on soil and plant health [1]. Moreover, some fertilizers contain heavy metals that can accumulate in the soil and enter the food chain through plants [2,3,4,5]. For these reasons, the study of alternative fertilizers, natural bio-stimulants and amendments is a current topic [6,7]. For instance, agricultural bio-stimulants, i.e., substances or microorganisms, are applied to plants or soils to improve nutrient efficiency, environmental stress tolerance and crop quality. Free amino acid-based formulations are exploited for regenerative agriculture since they are able to optimize endogenous plant nitrogen assimilation and mitigate the impacts of abiotic stressors such as drought, salinity and thermal extremes. This can be achieved by acting directly as metabolic precursors and cellular osmoprotectants. In this context, amino acid hydrolysis represents an advanced biorefinery pathway compared to conventional chemical synthesis. This method enables the strategic upcycling of organic by-products, focusing specifically on PAPs, by converting these complex proteinaceous matrices into free amino acids and highly bioavailable signaling molecules. This approach lowers environmental disposal burdens and drives the transition toward a functional circular bioeconomy. However, as the diversity of alternative biological and organic agricultural inputs expands, evaluating their pathogen and biological safety has become a strict prerequisite to prevent environmental contamination and ensure food safety before large-scale application [8]. For commercial organic inputs, comprehensive microbiological screenings—including total microbial load and the monitoring of indicator organisms or human pathogens like *Escherichia coli* and *Salmonella*—are critical to guarantee compliance with regulatory frameworks and certify a high safety profile.

Against this background, the present study aimed to evaluate the effects of an organic fertilizer obtained from animal by-products (ABPs). ABPs are materials derived from animals, or parts of animals, that are not intended for human consumption, i.e., carcasses or parts of them, digestive tract content, manure, feathers, hair and even certain food waste [9,10]. In Europe, over 20 million tonnes of ABPs are produced every year from slaughterhouses, industries producing food for human consumption, and fallen stock from farms. If ABPs are not effectively utilized, a valuable source of potential revenue may be lost; moreover, the increasing cost of disposal of these products is sustained by industry. Then, the efficient use of these products is a challenge [11,12]. ABPs rendering is the process of converting animal by-products into safe and usable product material. Depending on their category, as defined by Regulation (EC) No 1069/2009 [9], ABPs can be managed through incineration, anaerobic digestion for biogas and energy recovery, composting, fertilizer production or processing into value-added products including biofuels, pet food, pharmaceuticals and cosmetics [13,14]. All these treatments present numerous critical issues, among them, the following: long processing times, use of chemicals, production of pollutants, sources of unpleasant odors, need for high temperatures and pressures [15]. To overcome these limits, in 2016, Concimi Biologici S.r.l. (Assemini (CA) Italy) proposed an organic fertilizer obtained through a transformation process based on radio frequency treatment (International patent no. WO2016/012986 A1 dated 28 January 2016). Compared to conventional ABPs valorization processes, this method offers advantages in terms of processing time, environmental sustainability and product quality. In this work the agronomic performance of the above-described ABPs-derived fertilizer, PAPs, was studied and compared to a commercial fertilizer. Based on the collected results, this derived organic fertilizer represents a valid alternative to synthetic fertilizers, and its production from ABPs is a promising example of circular economy principles applied to more sustainable agriculture.

## 2. Results and Discussion

To optimally grow and develop, plants require specific elements and compounds called essential nutrients. There are 16 essential nutrients, divided according to the quantity required by the plant: (i) primary nutrients or macro-nutrients, required in large quantities, such as carbon, hydrogen, oxygen, nitrogen, phosphorus, potassium, calcium, magnesium and sulfur; (ii) micro-nutrients or trace elements, required in minimal quantities, such as boron, chlorine, copper, iron, manganese, molybdenum and zinc [16]. Nitrogen is essential for the synthesis of amino acids, proteins and chlorophyll and promotes vegetative growth; phosphorus is essential for energy processes; potassium regulates water balance, while manganese, zinc, copper and boron are essential for photosynthesis, respiration, cell wall formation and growth [17,18]. If even one of these nutrients is missing, the plant cannot complete its life cycle.

Since plant growth requires a balanced supply of these metal ions, our experiments focused on identifying the concentration of PAPs that provided an adequate nutrient input. As a first step, PAPs material was chemically characterized to determine its elemental composition, with particular attention to the presence of macro- and micro-nutrients, and to evaluate its potential as organic fertilizer. Subsequently, growth trials were conducted at three levels:

I. Germination tests, to assess potential phytotoxicity and seed tolerance to the product;
II. Hydroponic cultivation tests, to monitor vegetative growth under controlled conditions;
III. Pot trials, to evaluate the fertilizer performance under agronomic conditions more closely resembling field applications.

**Figure 1:**
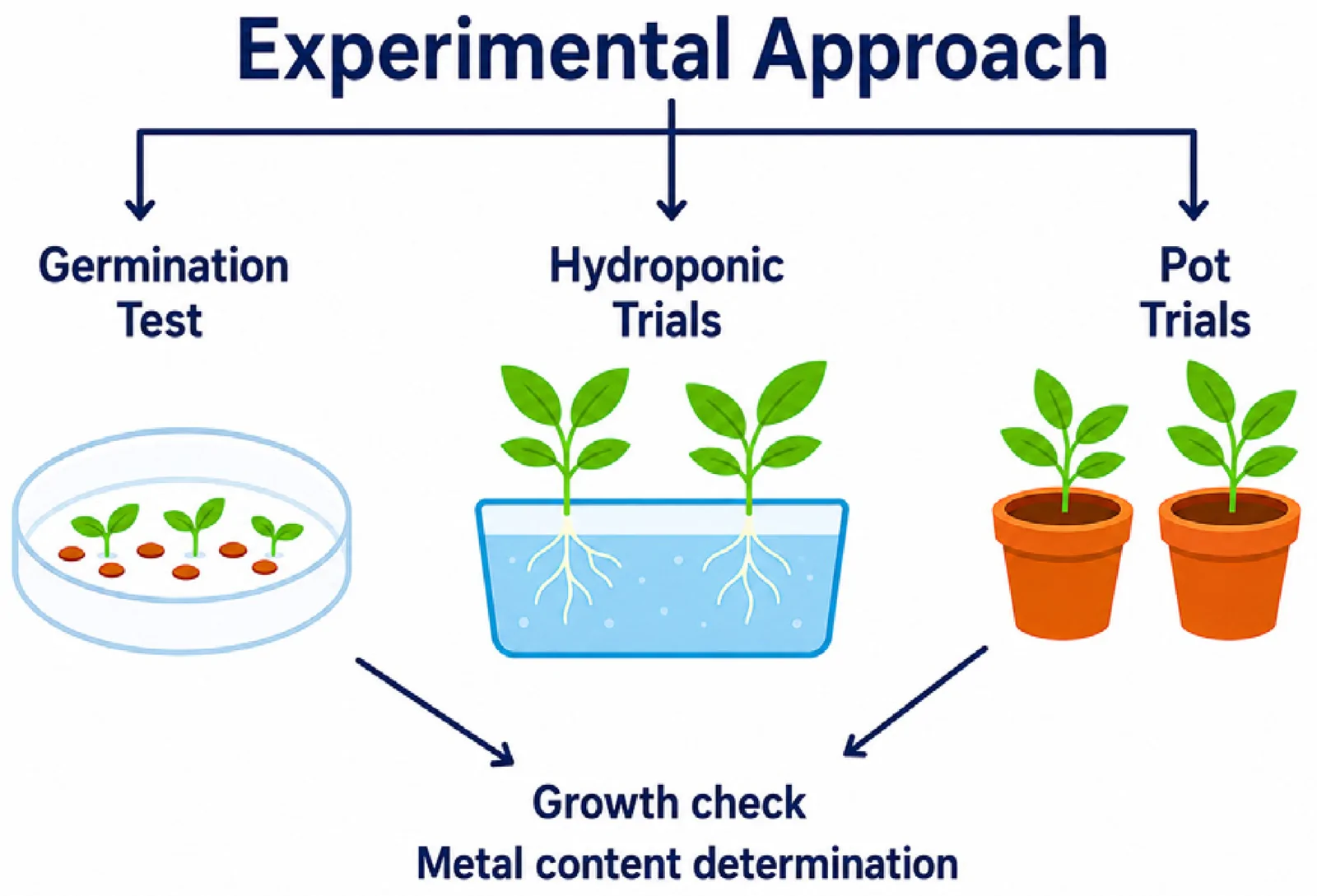
Experimental approach for the study of the PAP’s agronomic performance.

After fifteen days, the plants were harvested and analyzed for their micro- and macro-nutrient content. This experimental approach allowed us to correlate the chemical composition of the PAPs with the physiological responses of the plants (Figure 1). Overall, the results provided an assessment of the fertilizer’s efficacy and sustainability.

### 2.1. Characterization of PAPs 

**Figure 2:**
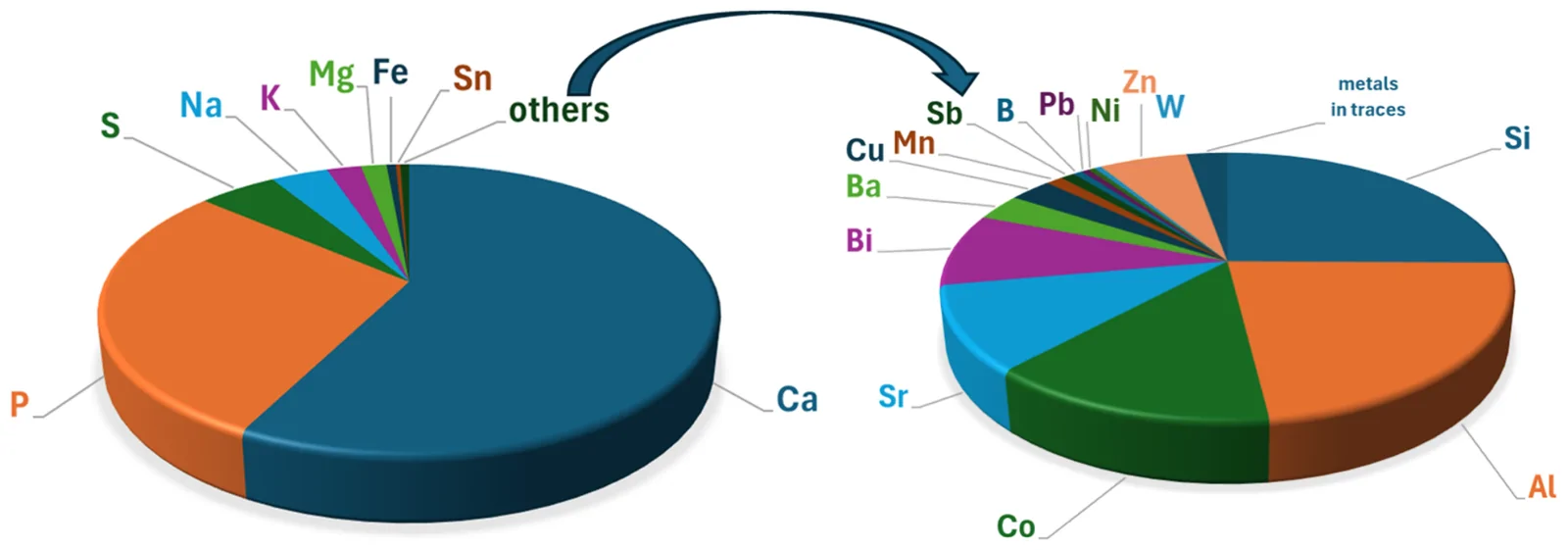
Elements present in PAPs at ≈80,000 − 800 mg kg^−1^ d.s. concentration range (**left**) and at ≈ 200 − 4 mg kg^−1^ d.s. (**right**); metals in traces are lower than 2 mg kg^−1^ d.s. (<0.01%).

The nitrogen and carbon content, soluble phosphorus, pH of aqueous suspension and metal/non-metal content of PAPs samples are reported in Table 1A,B. Metal content is also shown in Figure 2 for a visual comparison. The high content of calcium, phosphorus, potassium and sulfur confirms the agronomic potential of PAPs. These elements, essential for plant growth are, in fact, commonly found in synthetic fertilizers [19].

### 2.2. Regulatory Compliance Assessment

#### 2.2.1. Classification of Animal By-Products, Permitted Raw Materials and Processing Requirements

The raw materials used in this study comprise both Category 3 and Category 2 animal by-products as defined by Regulation (EC) No. 1069/2009 [9]. Category 3 materials originate from slaughtered animals considered fit for slaughter for human consumption, and are derived from slaughterhouses, tuna processing plants, butcher shops and large-scale retail distribution. In addition, the process receives fallen stock from pigs and poultry, which is classified as Category 2 material under the same Regulation (EC) No. 1069/2009 [9]. For the raw materials used in this study, the main regulatory concern is therefore related to biological and sanitary risks. These materials were processed using a microwave-based treatment (Fasthum technology) combined with steam, in accordance with the processing methods specified in Annex IV of Regulation (EU) No. 142/2011 (Methods 4 and 7) [28]. Microbiological analyses performed during the 30-day validation period on numerous aliquots of the processed PAPs demonstrated the effectiveness of the treatment in reducing the microbial load, particularly *Salmonella*, Enterobacteriaceae and *Clostridium perfringens*. The PAPs were also marked with the GHT tracer at a concentration of 250 mg kg^−1^ of fat, as required by Regulation (EU) No. 142/2011 [28].

#### 2.2.2. Pathogen Requirements and Microbiological Safety 

Microbiological evaluations confirmed the high sanitary quality and strict biological safety of the PAPs. Crucially, the presence of major regulatory pathogens and hygiene indicator organisms was entirely excluded across all analyzed production batches. Testing according to CEN/TS 17780:2022, UNI EN ISO 21528-2:2017, and ISO 15213-2:2023 standards confirmed the complete absence of *Salmonella* spp. (detected as absent in 25 g of product) [29,30,31]. Furthermore, both Enterobacteriaceae counts, and *Clostridium perfringens* levels were not detected, remaining strictly below the method’s limit of quantification (<10 CFU/g, with LOQ = 10 for both parameters). These findings demonstrate that the radio frequency-based transformation process effectively sanitizes the matrices, successfully delivering pathogen-free PAPs and eliminating the potential biological risks associated with the raw animal by-products. 

#### 2.2.3. Heavy Metal Limits and Fertilizer-Product Compliance

The heavy metal content in PAPs was compared with current regulatory thresholds to assess compliance with both European and national regulations. Specifically, the data were assessed in relation to Regulation (EU) 2018/848 [32], which governs the production, labeling and permissible inputs for organic farming, and to Regulation (EU) 2019/1009 [33], the benchmark European text which rules contaminant limits for CE-marked organic and mineral fertilizers. Furthermore, compliance was evaluated against the Italian National Legislation on fertilizers, namely, Legislative Decree No. 75 of 29 April 2010, with specific reference to the register of fertilizers permitted in organic agriculture outlined in Annex 13 (Table 2) [34]. Moreover, heavy metals are related primarily to the compliance requirements of the final fertilizer product rather than to the principal sanitary hazard associated with these ABPs. In this regard, Commission Implementing Regulation (EU) 2021/1165, Annex II, which lists fertilizers and soil conditioners authorized for use in organic farming, includes meat meal, bone meal, blood meal and fish meal among the permitted animal-derived materials [35]. The same Annex specifies that hexavalent chromium [Cr(VI)] must not be detectable for fur-derived materials; this restriction is therefore specifically associated with that material and does not apply to meat meal, bone meal, blood meal or fish meal.

The elemental analysis of PAPs revealed that the concentrations of the most important heavy metals were below the thresholds established by both Regulation (EU) 2019/1009 and (EU) 2021/1165) [33,35]. Specifically, key toxic elements such as cadmium (Cd ≤ 0.01 mg kg^−1^ d.s.) and inorganic arsenic (As ≤ 4 mg kg^−1^ d.s.) were near or below the detection limits, while lead (Pb) and total chromium (Cr) were present at safe levels of 4.3 (±0.9) mg kg^−1^ and 1.86 (±0.03) mg kg^−1^, respectively. The concentration of micro-nutrients like copper (Cu) and zinc (Zn) was lower than the European limits. An exception was observed for mercury (Hg), which was present at 1.3 ± 0.1 mg kg^−1^ and exceeded the regulatory limit of 1.0 mg kg^−1^ d.s. established by both European and Italian legislation. The Hg concentration measured in the PAPs is plausibly related to the fish-derived fraction of the raw materials. This is consistent with EFSA’s evidence [36] that methylmercury accumulates in the food chain and is found at higher concentrations in large predatory fish, particularly tuna and swordfish. It is remarked that regulation (EU) 2023/915 establishes a maximum Hg level of 1.0 mg kg^−1^ fresh weight for tuna and swordfish [37]. Considering that fresh tuna contains approximately 68–71% water, this corresponds to approximately 3.1–3.4 mg kg^−1^ on a dry-matter basis. In contrast, Regulation (EU) 2019/1009 establishes a maximum Hg concentration of 1.0 mg kg^−1^ dry matter for organic fertilizers (PFC 1(A)) [33]. Thus, the PAPs value reported in this work slightly exceeds the fertilizer limit, while remaining below the dry-matter equivalent of the maximum level established for tuna. This comparison is not intended to equate the two regulatory frameworks but highlights the markedly different thresholds applied to food and fertilizing products. Finally, Hg concentration should be considered together with the actual application rate. At the intended PAPs application rate of approximately 0.5–1 t ha^−1^, 1.3 mg kg^−1^ Hg corresponds to only 0.65–1.3 g Hg ha^−1^. By comparison, an organic amendment containing 0.4 mg kg^−1^ Hg and applied at 10–30 t ha^−1^ would introduce approximately 4–12 g Hg ha^−1^. Therefore, contaminant concentration alone does not fully describe the potential environmental load; the amount of product applied per hectare should also be considered.

### 2.3. Germination Tests 

**Figure 3:**
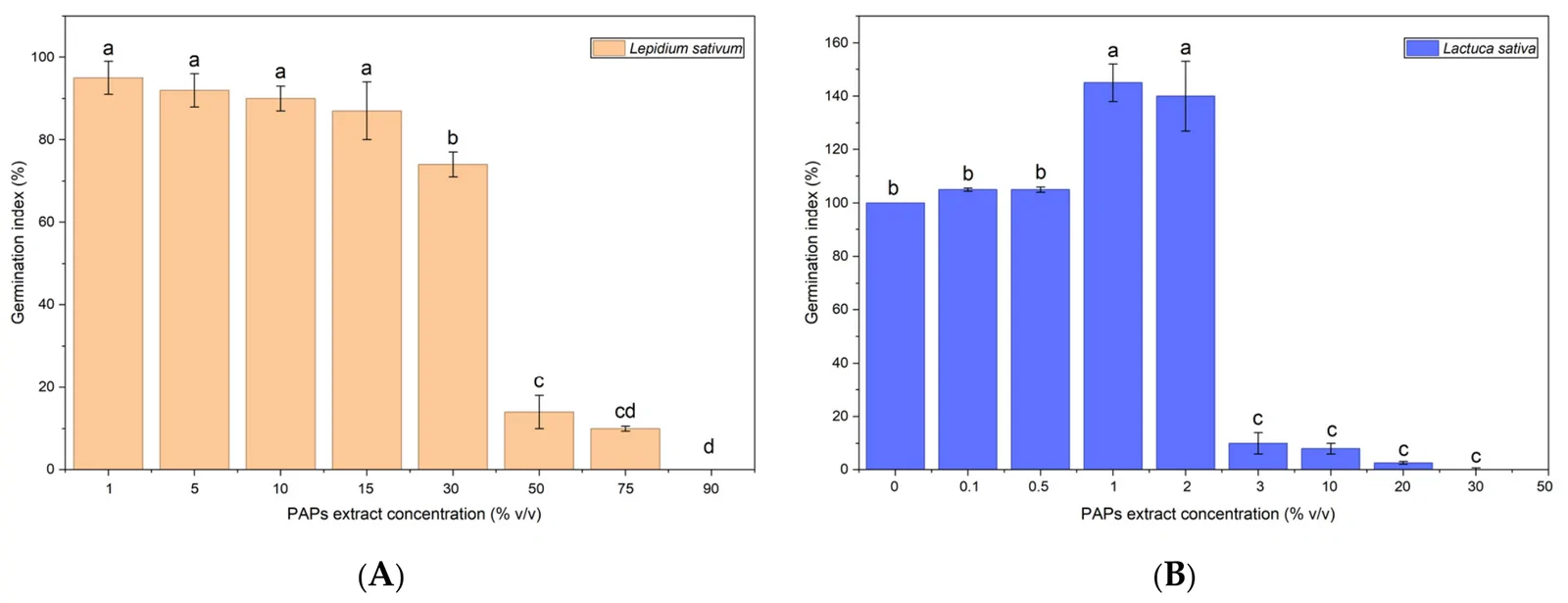
Germination index (GI) of *Lepidium sativum* (**A**) and *Lactuca sativa* (**B**) exposed to increasing PAPs concentrations. Low-to-moderate PAPs contents (0.1–2%) promoted germination, particularly in *Lactuca sativa*, whereas higher concentrations progressively inhibited germination, indicating phytotoxic effects. Solutions were prepared as reported in Section 4.1. Systems with 0 GI % are not plotted. Bars represent mean GI % and error bars indicate the standard deviation of measurements obtained from three independent Petri dishes (*n* = 3). Tukey letters are also shown (treatments sharing at least one letter are not significatively different).

Germination tests (GI) were carried to evaluate the phytotoxicity of PAPs material and its highest amount to be used for hydroponic cultivation. This test lasts 24 h and it is an economical and time-efficient method to detect simultaneously the effect on germination and root elongation. Different effects were observed with *Lepidium sativum* and *Lactuca sativa*. For *Lepidium sativum*, the PAPs-containing material was non-phytotoxic up to a concentration of 2% (see Section 4.1 for details), with the germination index showing a gradual decrease as the PAPs content increased. In contrast, *Lactuca sativa* exhibited a biostimulant response over the 0.1–2% concentration range, reaching a maximum GI of 145% at 1% PAPs. At higher PAPs concentrations, the material became phytotoxic for both species. Overall, these results identify an optimal PAPs concentration range of 1–2%, where the material promotes seed germination and early growth without inducing phytotoxic effects (Table 3 and Table 4, Figure 3 and Figure S1, Sections S1 and S2).

### 2.4. Hydroponic Cultivation Tests

Several trials were conducted with *Lactuca sativa* and *Lepidium sativum*, varying the concentration of PAPs alone or in the presence of a commercial nutrient solution (C) formulated to ensure an optimal nutrient balance. The commercial nutrient solution (two parts to be combined) provides a balanced supply of macro- and micro-nutrients for optimal plant growth (Part A: total nitrogen N 3.4%, N-NH4 0.3%, nitric nitrogen N-NO_3_ 3.1%, CaO 2.1%, Fe-DTPA complex 0.02%, Fe-EDDHA complex 0.03%, Mn-DTPA complex 0.01%, electric conductivity variable from 1.3 to 2.1 mS cm^−1^; part B: water soluble P_2_O_5_ 3.7%, water soluble K_2_O 9.4%, MgO 1.7%, SO_3_ 4.3%, B 0.01%, Cu 0.001%, Mn 0.018%, Mo 0.002%, Zn 0.01%,). 

**Figure 4:**
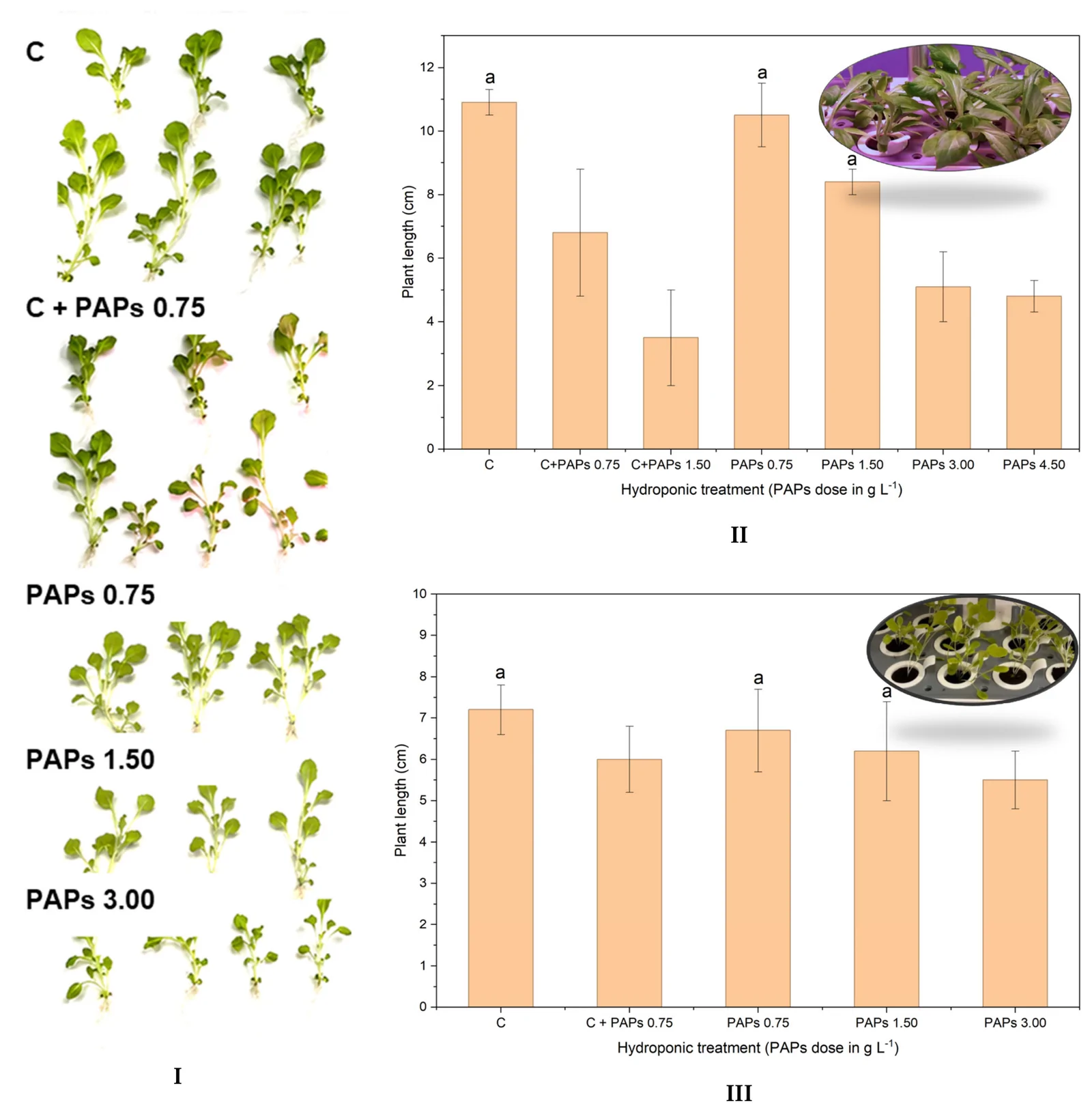
Growth of *Lactuca sativa* (**I**,**II**) and *Lepidium sativum* (**III**) on the tested systems, at different PAPs content (g L^−1^), in the presence of commercial nutrient solution (C) or in the presence of commercial nutrient solution and PAPs at 0.75 or 1.50 g L^−1^; for C, PAPs 0.75 and PAPs 1.50 g L^−1^, bars represent means ± SD of two independent tank−level means. For the remaining treatments, bars represent means ± SD of 12 plants from a single tank. The one−way ANOVA test applied on tank−level mean plant lengths (two independent tanks par treatment) did not show statistically significant differences among C, PAPs 0.75 g L^−1^, and PAPs 1.50 g L^−1^ for *Lactuca Sativa* (F value = 6.43; *p* = 0.0822) and for *Lepidium Sativum* (F value = 0.2679, *p* = 0.781). Tukey letters are reported only for replicated treatments; shared letters indicate no significant difference according to Tukey’s HSD test (α = 0.05).

At first, an exploratory screening was performed using one independent hydroponic tank for each treatment. Since PAPs concentrations above 3 g L^−1^ did not support plant growth, these systems were not replicated, while the control and 0.75 and 1.50 g L^−1^ PAPs-containing tanks, were subsequently repeated using a second independent tank. Thus, these three systems comprised two independent tanks (*n* = 2), whereas the reinforced control and the remaining PAPs treatments were evaluated in one tank (*n* = 1). Each tank contained 12 sowing stations with five seeds per station. Because the stations shared the same hydroponic solution, they were considered subsamples rather than independent biological replicates. After 15 days, the seedlings were harvested, and the total plant length—comprising both root and shoot—was measured (Figure 4, Table 5). In all the trials, the control treated with the nutrient solution showed the greatest growth. Reinforced control, i.e., systems containing both nutrient solution PAPs (0.75 g L^−1^) and commercial nutrient solution, showed lower growth levels than treatments with only 0.75 g L^−1^ or 1.50 g L^−1^ of PAPs. The lowest growth levels were observed in samples treated with 3.00 g L^−1^ and 4.50 g L^−1^ of PAPs. The 0.75 g L^−1^ of PAPs treatment resulted in growth levels comparable to those of the control. Greater concentrations of PAPs (9.00 g L^−1^) were ineffective for growth and were not further tested.

The ANOVA test applied on tank-level mean plant lengths (two independent tanks per treatment) did not show statistically significant differences among C, PAPs 0.75 g L^−1^ and PAPs 1.50 g L^−1^ for both the plants (F-value = 6.43; *p* = 0.0822) for *Lactuca Sativa*; F = 0.2679, *p* = 0.781 for *Lepidium Sativum*). These findings, that at first may appear disappointing, suggest instead that the animal-derived by-product PAPs may support plant growth in a way similar to that observed with the commercial nutrient solution C. Nevertheless, the limited replication prevents a conclusion of equivalence. Among the tested PAPs concentrations, 0.75 g L^−1^ appears preferable, as it required half the material content, even yielding a mean plant length closer to that of the commercial control. ANOVA results are detailed in the Supplementary Materials (Sections S3 and S4).

**Figure 5:**
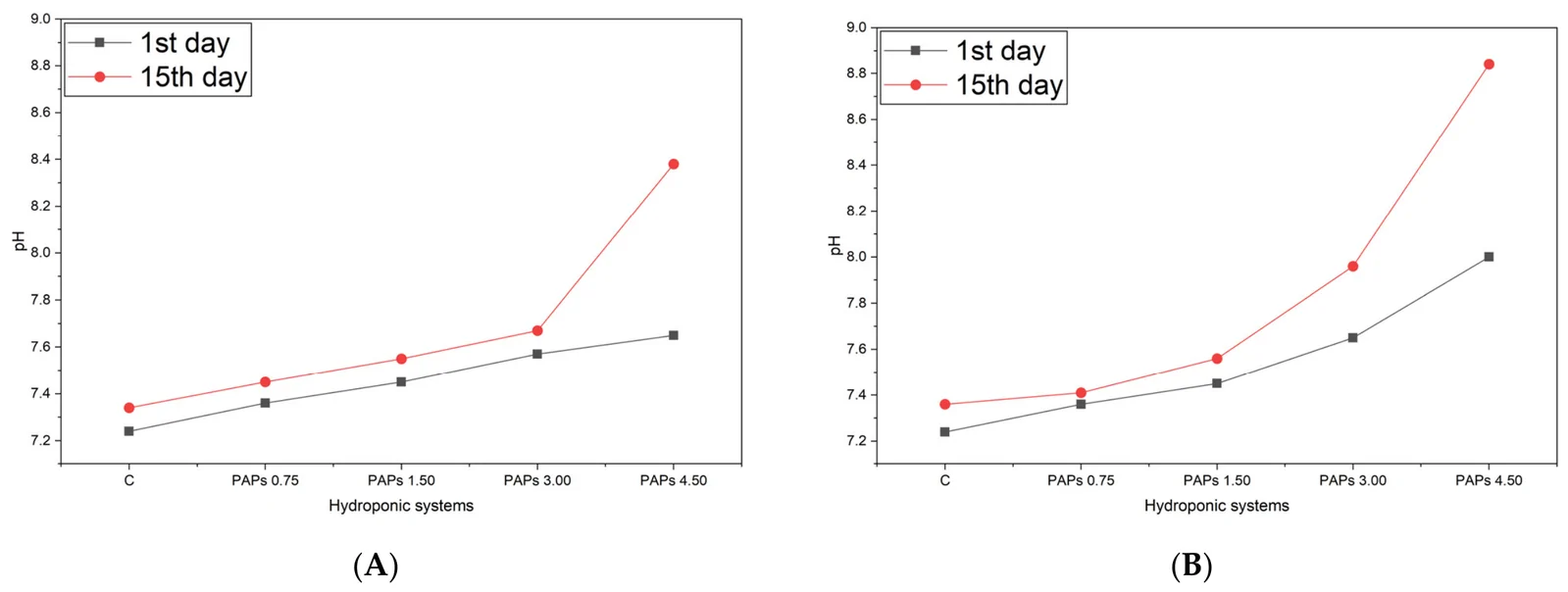
pH changes occurring during 15 days for the studied hydroponic systems, (**A**) *Lactuca sativa* and (**B**) *Lepidium sativum* at different PAPs content (g L^−1^) and in the presence of commercial nutrient solution (C). For each hydroponic tank, pH measurements were performed in technical triplicate. The coefficient of variation among the replicate measurements was ≤5%.

Since pH is an important parameter influencing solubility and adsorption of nutritional elements, hydroponic solution pH was monitored for 15 days for all the systems (Figure 5). The pH values naturally slightly increased during the time (from 7.23 to 7.34, + 1.4%), but relevant pH increase was observed for systems with high PAPs concentration, in particular, at 3 g L^−1^ for *Lactuca sativa* (from 7.65 to 8.38, +9.5%), and already at 2 g L^−1^ for *Lepidium sativum* (from 7.65 to 7.96, +4.1%) and more greatly at 3 g L^−1^ (from 8.00 to 8.84, +10.5%).

### 2.5. Pot Culture Tests

**Figure 6:**
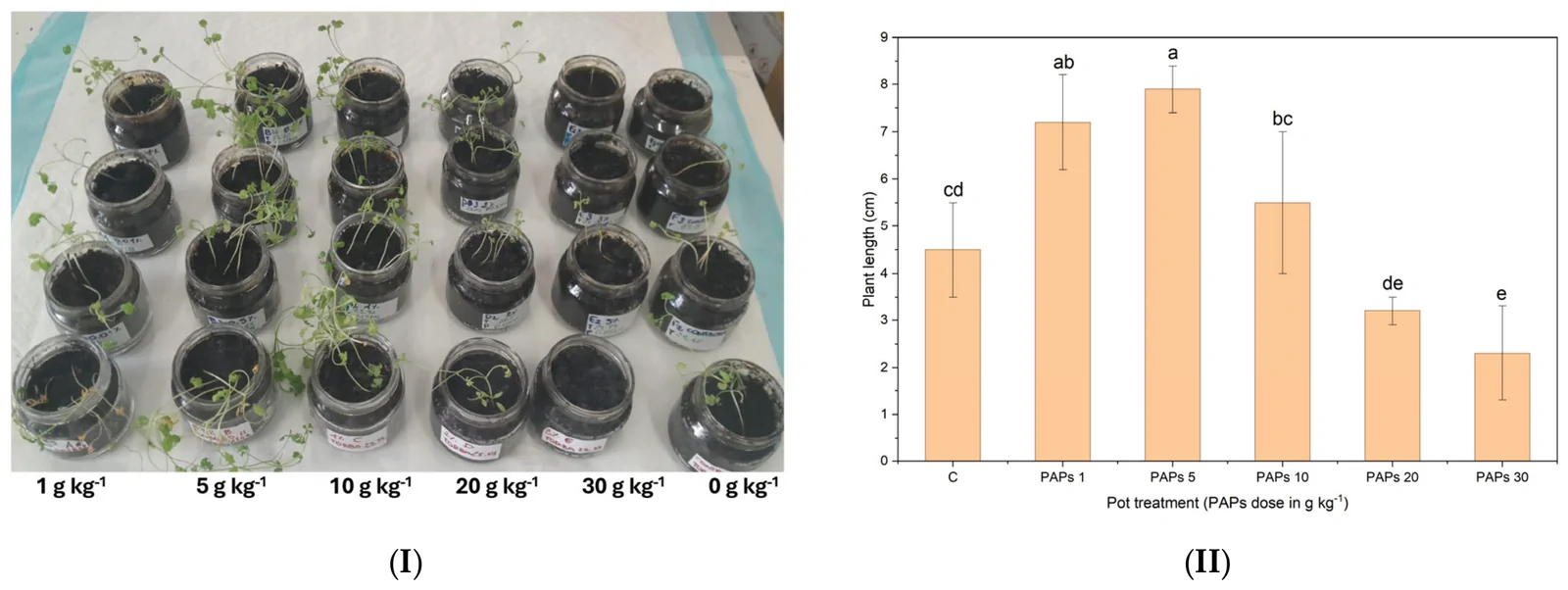
*Lepidium sativum* in pots tests (**I**) and corresponding growth (**II**) at different PAPs concentration (from 1 g kg^−1^ to 30 g kg^−1^). Four independent pots were prepared for each treatment (*n* = 4), with five seeds per pot. Bars represent the mean of the four independent pot−level means, and error bars indicate the standard deviation among the pot−level means. C refers to the control pots with only peat moss. Tukey letters are shown. Treatments sharing at least one lowercase letter are not significantly different according to Tukey’s HSD test (α = 0.05).

Pot tests were carried out on *Lepidium sativum*, testing systems with PAPs at 1 g kg^−1^, 5 g kg^−1^, 10 g kg^−1^, 20 g kg^−1^ and 30 g kg^−1^. Control pots (C) contained only peat moss. All pot experiments were performed using four independent pots per treatment (*n* = 4). Each pot contained five seeds and was considered an independent biological replicate, whereas the plants grown within the same pot were considered subsamples. Additions of PAPs till 5 g kg^−1^ of concentration improved the plant growth with respect to the controls. PAPs concentrations higher than 5 g kg^−1^ resulted in an evident decrease in growth, causing a progressive decline in plant development (Figure 6). Plants treated with 5 g kg^−1^ PAPs showed the best growth; thus, this concentration appears the most suitable level among those tested, promoting plant development. The one-way ANOVA test on pot−level mean plant lengths showed a significant treatment effect (*n* = 4 independent pots per treatment; F-value = 31.81, *p* < 0.001; see Supplementary Materials Section S5). In fact, Tukey’s HSD test provided evidence that PAPs at 1 and 5 g kg^−1^ significantly increased plant length compared with the control (peat moss), while higher PAPs content, such as 30 g kg^−1^, negatively affected plant growth in a significant way (Table 6). The most favorable dose was 5 g kg^−1^, as evidenced before, although this treatment did not differ significantly from 1 g kg^−1^.

### 2.6. Metal Ion Content in Plant Samples 

#### 2.6.1. Hydroponic Systems

**Figure 7:**
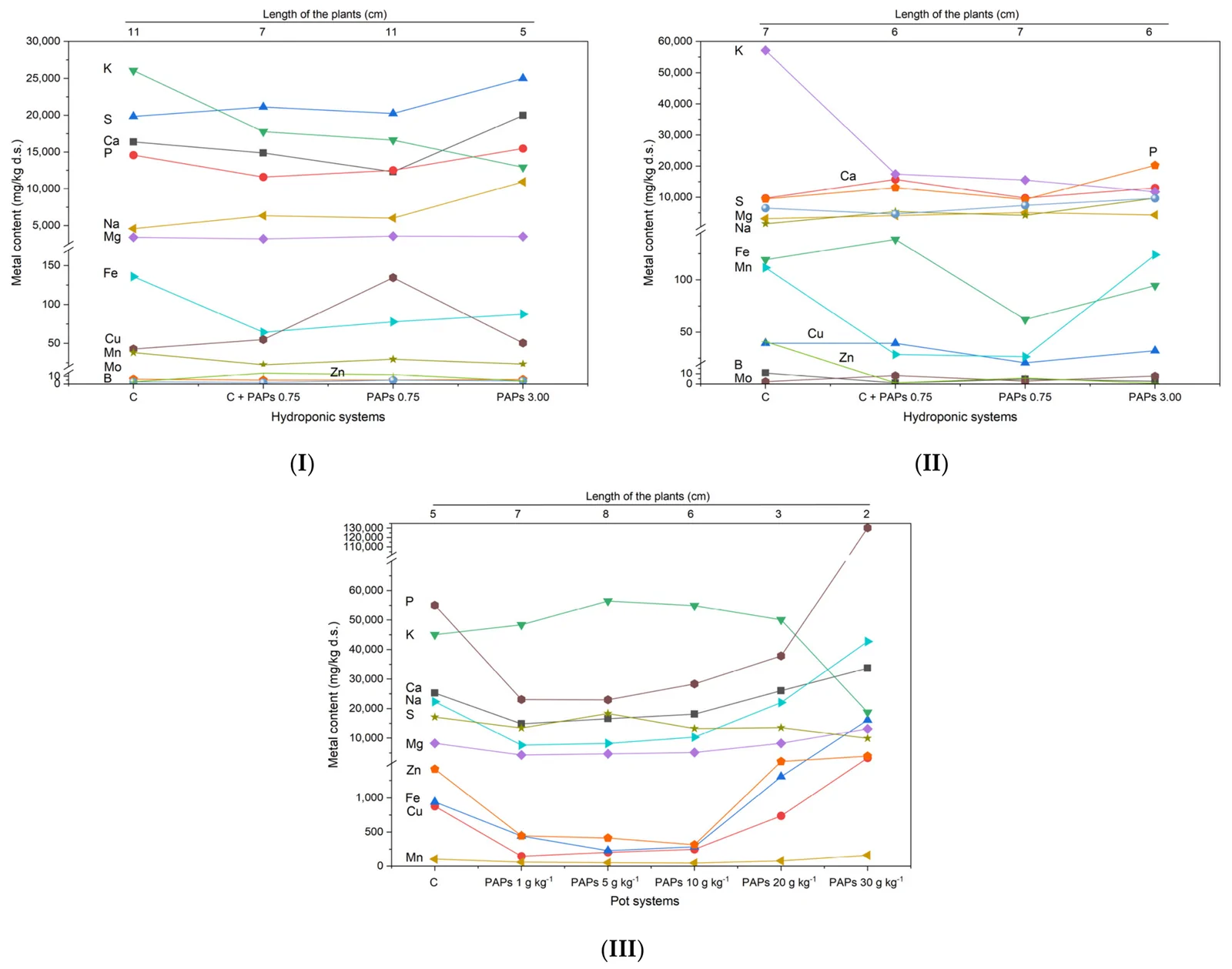
Metal ion content (mg kg^−1^ dried sample) in *Lepidium sativum* (**I**) and *Lactuca sativa* (**II**) cultivated in hydroponic systems under different experimental conditions (C commercial nutrient solution, PAPs content is expressed in g L^−1^); for *Lepidium sativum* (**III**) under different experimental conditions (C, peat moss topsoil) in pot cultivation (g kg^−1^). In the upper part of the graph, the mean length values of the plants are reported (see Figure 4 and Figure 6 for details).

*Lepidium sativum* plants treated with 0.75 g L^−1^ PAPs showed lower Ca and P concentrations than those observed in plants treated with the commercial nutrient solution (Ca −25%, P −15%), while S and Mg contents were similar (Figure 7I). In contrast, the potassium supply provided by PAPs in hydroponic systems was lower than that provided by the commercial solution (−36%). As regards micro-nutrients, Fe was depleted (−43%) while Cu was markedly increased (+212%); Mn, B and Zn showed variations from 38 to 30 mg kg^−1^, from 3 to 5 mg kg^−1^, and from 3 to 12 mg kg^−1^, respectively. At PAPs concentrations higher than 0.75 g L^−1^, most metal ions were present in plants at higher concentrations than in plants grown with the commercial nutrient solution alone; however, as explained above, plant growth was negatively affected under these conditions.

These results suggest that an excess of micro-nutrients may contribute to a phytotoxic effect. Therefore, 0.75 g L^−1^ PAPs appear to be the most suitable concentration under the tested conditions, while 4.50 g L^−1^ should be avoided since it resulted in adverse effects on plant growth. Moreover, the combined administration of the nutrient solution and PAP_S_ at 0.75 g L^−1^ resulted in increased nutrient concentrations in plant tissues for most metal ions compared with each treatment alone, except for K, Ca, Fe and Mn. This may be explained by alteration in the ion absorption dynamics, potentially driven by competitive interaction among the ions present in the PAP_S_ and those in the nutrient solution, which may have altered the availability of specific elements and enhanced or reduced root uptake. Consistently, plants subjected to the combined treatment showed poorer growth than those treated with either the nutrient solution or PAPs alone. 

Regarding *Lactuca sativa*, plants treated with 0.75 g L^−1^ PAP_S_ showed Ca and P concentrations similar to those observed with the commercial nutrient solution (Ca +0.5%, P −1.6%), while the S and Mg content increased (+13% and + 60%, respectively) (Figure 7II). As observed with *Lepidium sativum*, K content markedly decreased (−73%), indicating that the potassium supplied by PAPs in hydroponic systems was lower than that supplied by the commercial solution. Regarding micro-nutrients, almost all analyzed metal ions were depleted when the plants were treated with PAPs at 0.75 g L^−1^ (Fe −48%, Cu −33%, Mn −77%, B −55% and Zn −85%). The same trend was observed with PAPs at 3.00 g L^−1^, with the exception of Mn, which increased by +11%. The combined administration of the nutrient solution and PAPs at 0.75 g L^−1^ resulted in increased concentrations of Ca, P, Fe and Mo, and decreased concentrations of K, S, Mn and Zn compared to each treatment alone. As observed for *Lepidium sativum*, altered absorption dynamics may be involved, consistent with the poor growth observed under this combined treatment. 

#### 2.6.2. Pot Systems

As can be seen in Figure 7III, the addition of PAPs at concentrations 1–10 g kg^−1^ to the peat moss topsoil leads to decrease in almost all the micro- and macro-nutrients, except for potassium; on the contrary, at higher PAPs concentrations, the metal content is greatly increased, especially for phosphorus. However, the growth of the plants was in the order PAPs 5 g kg^−1^ > PAPs 1 g kg^−1^ > PAPs 10 g kg^−1^ > C > PAPs 20 g kg^−1^% > PAPs 30 g kg^−1^. 

#### 2.6.3. Selected Experiments

**Figure 8:**
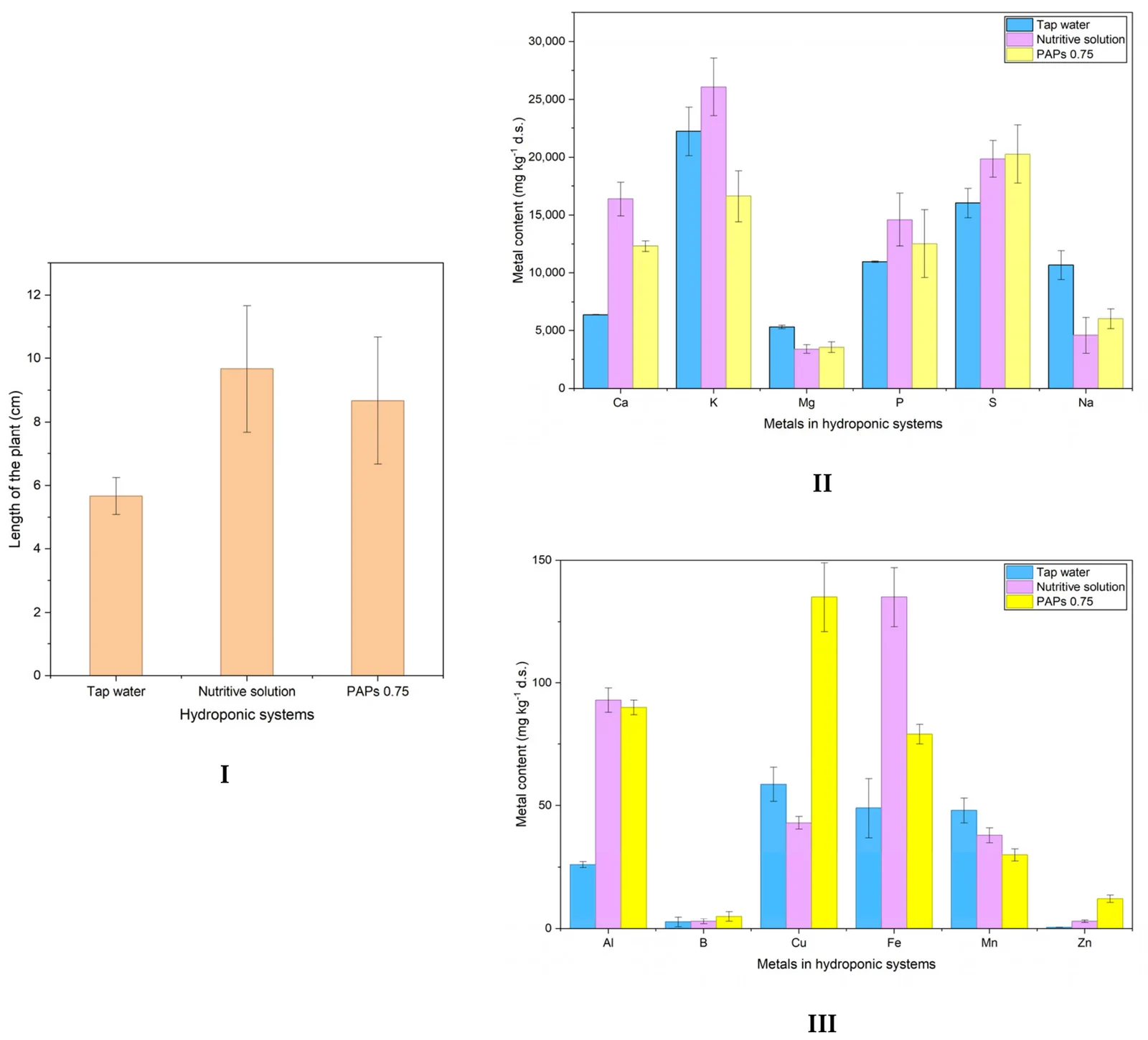
Growth (**I**) and metal ion content (**II**,**III**) of *Lepidium sativum* cultivated in hydroponic system with tap water, in the presence of commercial nutrient solution or with PAPs at 0.75 g L^−1^. Each treatment was represented by one hydroponic tank. In (**I**), bars represent the mean length of 12 plants per tank, and error bars indicate the standard deviation among individual plants. For elemental analysis, the dried plant material from the 12 plants harvested from each tank was divided into three aliquots and digested separately. In (**II**,**III**), bars represent the mean metal ion content measured in the obtained three aliquots, error bars indicate their standard deviation. Results are reported in a descriptive way, without inferential statistical comparisons among treatments.

To further compare the effects of PAPs on plant performance, additional experiments were carried out with *Lepidium sativum* in tap water, or in the presence of the commercial nutrient solution or with 0.75 g L^−1^ PAPs. The concentration of the main nutrients of interest for plant growth (macro-, meso- and micro-nutrients) in plant tissue under the various experimental conditions are summarized in Figure 8. As can be observed, plants grown with 0.75 g L^−1^ PAPs showed metal concentrations and growth comparable to those grown with the commercial nutrient solution. In contrast, plants grown in tap water alone showed lower ion concentration and poorer growth compared to those treated with either PAPs or nutrient solution.

## 3. Conclusions

The results obtained in this study indicate that PAPs material represents a viable source of both macro- and micro-nutrients, capable of supporting plant growth and nutrient uptake. From an industrial perspective, this matrix offers a dual economic advantage: it may reduce waste disposal costs while promoting the valorization of a processing residue into a potentially marketable by-product. Its effectiveness is closely related to both the concentration of PAPs applied and the cultivation system adopted, highlighting the importance of carefully selecting the most appropriate concentration.

In the tested hydroponic systems, moderate PAPs concentrations (0.75–1.50 g L^−1^) supported plant development and nutrient uptake, whereas higher PAPs concentrations resulted in growth inhibition, which may be associated with nutrient imbalance and/or excessive accumulation of specific elements. Most likely, the growth inhibition observed at the highest PAPs concentrations was multifactorial. In fact, in addition to changes in elemental concentrations and possible ionic interactions, the marked increase in solution pH may have altered nutrient speciation, solubility and availability, potentially limiting plant uptake. Because PAPs concentration and pH increased concomitantly, their individual contributions cannot be distinguished. It is important to note that in the control tanks, the pH values remained essentially stable throughout the experiment, whereas PAPs-containing systems showed a concentration-dependent alkalinization. The largest pH increases occurred at the highest PAPs concentrations, which were also associated with the poorest plant growth.

When PAPs were applied together with the commercial nutrient solution, no additive effect on plant growth was observed. This response may be related to altered ion availability and/or competitive interactions affecting nutrient uptake; however, these mechanisms were not directly investigated in the present study. 

In pot trials, PAPs exhibited more stable behavior. Plants treated with PAPs at concentrations 1, 5 and 10 g kg^−1^, showed good growth rates compared to the control, while higher concentrations progressively inhibited plant development. These results indicate that an appropriate ionic balance is important for plant development and that an imbalanced nutrient supply can reduce or inhibit plant growth.

In conclusion, based on the regulatory comparative analysis, PAPs show promising potential as a sustainable alternative nutrient source. Through the recovery and valorization of by-products, this material may represent a valuable candidate within the framework of organic farming, in line with circular economy principles. Overall, the present study should be regarded as an early-stage proof-of-concept, demonstrating the potential of PAPs under controlled experimental conditions rather than as complete agronomic validation. Further larger-scale and field studies are needed to confirm their performance, optimal application rates and suitability across different crops and cultivation conditions.

The primary aim of this study was to establish an experimental framework for studying the agronomic valorization of processed animal protein by-products, identify potentially suitable application ranges, and determine plant responses to be examined in subsequent validation studies. The results should therefore be read as preliminary findings that identify trends, methodological priorities, suitable experimental conditions and critical points to be addressed for future studies, employing fully replicated designs. Overall, these findings indicate that, under the tested experimental conditions and at appropriate concentrations, PAPs have the potential to serve as an alternative nutrient source. However, further studies are required before their fertilizer performance can be directly compared with that of commercial nutrient solutions.

## 4. Materials and Methods

*Lactuca sativa* L. ‘Laibacher Eis’ and *Lepidium sativum* L. plants were grown from commercially purchased seeds. The *L. sativa* seeds were marketed by Magic Garden Seeds GmbH (Regensburg, Germany), and the *L. sativum* seeds were marketed under the ZENGREENS brand. No plant material was collected from wild populations.

### 4.1. Germination Tests

Germination tests (GI) were carried out according to the section “Germination tests, microbiological tests for compost analysis” of the APAT method [38]. A total of 37.5 mL of distilled water was added to 12.5 g of PAP_S_. After 2 h, the mixture was centrifuged at 6000 rpm for 15 min, and the resulting supernatant was filtered. The filtered supernatant was considered the undiluted extract (100%, *v*/*v*) and was subsequently diluted with distilled water to obtain the desired extract concentrations, ranging from 1% to 75% (*v*/*v*). For example, the 1% (*v*/*v*) solution was prepared by diluting 100 µL of the filtered supernatant to a final volume of 10 mL with distilled water. One mL aliquots of the resulting solutions were collected and placed in a Petri dish lined with filter paper. Ten plant seeds were allowed to swell for 1 h in distilled water and then added to each dish and incubated for 24 h. Water-only controls were also prepared. For each treatment, three Petri dishes were used, and each Petri dish was considered an independent biological replicate. At the end of the incubation, the germinated seeds were counted and the root length measured. The Germination Index (Ig) was calculated as follows:

Ig % = (Gc × Lc/Gt × Lt) × 100

where Gc is the number of germinated seeds in the sample, Gt is the number of germinated seeds in the control, Lc is the mean root length in the sample and Lt is the mean root length in the control. The germination index was calculated separately for each Petri dish, and the mean germination index for each treatment was then obtained by averaging the values across the biological replicates.

### 4.2. Hydroponic System Tests

Seeds of *Lactuca sativa* and *Lepidium sativum* at different PAPs concentrations were grown. 

The PAPs stock suspension was prepared by placing 15.0 g of PAPs in a 100 mL volumetric flask and bringing to volume with distilled water, corresponding to a nominal concentration of 150 g L^−1^. Volumes of 15, 30, 45, 60, 75 and 90 mL of this stock suspension were added to the hydroponic tanks, and each tank was then brought to a final volume of 3.0 L. These treatments therefore corresponded to nominal final PAPs concentrations of 0.75, 1.50, 2.25, 3.00, 3.75 and 4.50 g L^−1^, respectively. Two additional systems were included. The control (C) contained 30 mL of the commercial nutrient solution supplied with the hydroponic kit and was brought to a final volume of 3.0 L with water. The reinforced control contained 30 mL of the same commercial nutrient solution together with 15 mL of the PAPs stock suspension and was brought to a final volume of 3.0 L with water, corresponding to a nominal PAPs concentration of 0.75 g L^−1^.

Each hydroponic system consisted of 12 sowing stations, with five seeds per station. Plants were collected after 15 days of growth. 

### 4.3. Pot Culture Tests

Transparent pots with a diameter of 5 cm were used for the pot culture tests. Each pot contained 25 g of peat and different concentrations of PAPs to reach 1 g kg^−1^, 5 g kg^−1^, 10 g kg^−1^, 20 g kg^−1^ and 30 g kg^−1^. The control consisted only of peat without PAPs. All tests were performed in quadruplicate. The contents of each pot were homogenized, and 10 mL of tap water was added. Five seeds of *Lepidium sativum* were placed in each pot. Water was added every 2–3 days. After 15 days of growth, plant samples were collected.

### 4.4. Quantifying Root-to-Bud Longitudinal Extension in Seedlings

To prevent tissue dehydration, morphometric measurements were performed on intact, fresh seedlings from both pot and hydroponic experiments immediately after collection. The specimens were aligned sequentially, and the total longitudinal extension—spanning from the root apex to the apical bud—was quantified using standard millimeter graph paper. Furthermore, leaf color was qualitatively evaluated through visual inspection. Additionally, serial photographic documentation was acquired to visually monitor and compare phenotypic development across the different experimental conditions.

### 4.5. Plant Samples Digestion 

The 12 plants of a tank were harvested, dried, finely ground and divided into three aliquots. Each aliquot (1.00 g) was transferred in a round-bottom flask with 2 mL of distilled water, 3 mL of 34.5% hydrogen peroxide and 2 mL of 45% nitric acid. The mixture was refluxed until complete plant digestion (approx. 20 min). The resulting solution was filtered and diluted to a volumetric flask (20 mL) and analyzed by atomic emission spectroscopy (ICP-OES) Agilent ICP-OES 5110 (Agilent Technologies Italia S.p.A., Via P. Gobetti 2/C, 20063 Cernusco sul Naviglio MI). When necessary, the solution was diluted to 1:10, 1:100 or 1:1000 with 2% HNO_3_ distilled water. Each sample was tested in a triplicate.

### 4.6. PAPs Digestion 

An aliquot of PAPs (0.50 g) was transferred in a round-bottom three-neck flask with 5 mL of distilled water and 5 mL of concentrated nitric acid (45%). The mixture was refluxed, then 5 mL more of nitric acid was added and, after 10 min, 5 mL of hydrogen peroxide (34.5%) was added, three times at 15 min intervals. Concentrated hydrochloric acid (10 mL, 37%) was added and the resulting mixture was refluxed for two hours. After cooling, the digestate was filtered and collected in a volumetric flask (50 mL). When necessary, the solution was diluted to 1:10, 1:100 or 1:1000 with 2% HNO_3_ distilled water. Each sample was tested in a triplicate.

### 4.7. ICP-OES Analysis

The metal and non-metal ions content was determined with an Agilent ICP-OES 5110 (Agilent Technologies Italia S.p.A., Cernusco sul Naviglio, Milan, Italy) equipped with an autosampler. Silver (Ag), aluminum (Al), arsenic (As), gold (Au), boron (B), barium (Ba), beryllium (Be), bismuth (Bi), calcium (Ca), cadmium (Cd), cerium (Ce), cobalt (Co), chromium (Cr), copper (Cu), europium (Eu), iron (Fe), allium (Ga), germanium (Ge), mercury (Hg), indium (In), potassium (K), lanthanum (La), lithium (Li), magnesium (Mg), manganese (Mn), molybdenum (Mo), sodium (Na), nickel (Ni), phosphorus (P), lead (Pb), platinum (Pt), sulfur (S), antimony (Sb), selenium (Se), silicon (Si), tin (Sn), strontium (Sr), titanium (Ti), vanadium (V), tungsten (W), yttrium (Y), zinc (Zn) were quantified. Starting from commercial ICP standards, for each element, 5 solutions were prepared with concentrations 0.01, 0.1, 1, 5 and 10 ppm for the calibration curves. Method detection limits (LODs) and method quantitation limits (LOQs) were calculated as three and ten times, respectively, the standard deviation of the procedural blank signal divided by the slope of the corresponding calibration curve. The calculated concentrations were referred to the initial dry sample mass and are reported as mg kg^−1^ dry weight. Analytical performance was monitored by analyzing a recovery sample after every 10 samples and by periodically reanalyzing selected calibration standards. Recoveries ranged from 99 to 101%, confirming the accuracy and stability of the analytical procedure. Procedural blanks were subjected to the entire analytical procedure, including digestion. Although no matrix-matched certified reference material was available for the PAPs and plant samples, the procedural blanks, recovery tests and periodic verification of the calibration standards provided quality-control checks throughout the analytical sequence.

## Figures and Tables

**Table 1:** (**A**) Chemical characterization of PAPs (the available characterization provided by the producer included total, soluble and ammoniacal N, whereas further N speciation, including nitrate-N, amino-acid composition and direct quantification of the organic-N fraction, was not available). (**B**) Metal/non-metal content of PAPs.

(**A**)		
**Element**	**Value**	Analytical Procedure *
Nitrogen (% N)	8.0 ± 0.5	UNI EN 1482-2:2007 + UNI EN 15604:2009 P.TO 8.1 [20,21]
Ammoniacal nitrogen (% N-NH_3_)	0.10 ± 0.05	UNI EN 15604:2009 P.TO 8.2.5 [21]
Soluble nitrogen (% N)	2.2 ± 0.4	UNI EN 15604:2009 P.TO 8.2 [21]
Soluble phosphorus in acidic media (% P_2_O_5_)	11 ± 1	UNI EN 1482-2:2007 + UNI EN 15956:2012 + EN 15959:2023 [20,22,23]
Soluble phosphorus in water (% P_2_O_5_)	<1.0	UNI EN 1482-2:2007+UNI EN 15958:2012+EN 15959:2023 [20,23,24]
Total organic carbon (%)	32 ± 3	DM 21/12/2000 GU N 21 26/01/2001 SUPPL 6 ALL [25]
Humic and fulvic carbon (%)	14 ± 1	DM 21/12/2000 GU n 21 26/01/2001 Suppl 6 All [25]
pH in water	6.4 ± 0.2	DM 17/06/2002 GU N°220 19/09/2002 SUPPL.7 MET III.3 [26]
Humidity (%)	5.7 ± 0.2	CEN/TS 17773:2022 [27]
(**B**)		
**Element**	**Concentration** **(mg kg^−1^ d.s.)**	**Relative Contribution to the Sum of Determined Elements (%) ^†^**
Calcium	80,746 ± 421	57.5
Phosphorus	40,361 ± 771	28.8
Sulfur	6909 ± 22	4.9
Sodium	5042 ± 102	3.6
Potassium	3016 ± 18	2.1
Magnesium	2266 ± 61	1.6
Iron	807 ± 5	0.6
Tin	393 ± 22	0.3
Silicon	209 ± 5	0.1
Aluminum	190 ± 1	0.1
Cobalt	115 ± 2	0.1
Strontium	85 ± 5	0.1
Bismuth	73 ± 4	0.1
Zinc	50 ± 1	0.04
Barium	26.8 ± 0.6	0.02
Copper	24 ± 2	0.02
Manganese	9.5 ± 0.1	0.01
Antimony	7.9 ± 0.6	0.01
Boron	5.2 ± 0.5	<0.01
Lead	4.3 ± 0.9	<0.01
Nickel	3.8 ± 0.2	<0.01
Tungsten	3.8 ± 0.7	<0.01
Arsenic	3 ± 1	<0.01
Platinum	≤3	<0.01
Gold	2.1 ± 0.2	<0.01
Titanium	2.1 ± 0.2	<0.01
Vanadium	≤2	<0.01
Chromium	1.86 ± 0.03	<0.01
Lanthanum	1.75 ± 0.1	<0.01
Selenium	1.55 ± 0.05	<0.01
Molybdenum	1.5 ± 0.5	<0.01
Mercury	1.3 ± 0.1	<0.01
Silver	1.13 ± 0.03	<0.01
Germanium	1.1 ± 0.1	<0.01
Gallium	1.06 ± 0.1	<0.01
Indium	≤0.2	<0.01
Beryllium	≤0.01	<0.01
Cadmium	≤0.01	<0.01
Cerium	≤0.01	<0.01
Europium	≤0.01	<0.01
Lithium	≤0.01	<0.01

* These analyses were performed by a certified analytical laboratory. ^†^ Relative elemental contribution (%) = C_i_/ΣC_j_ × 100.

**Table 2:** Heavy metal content in PAPs compared with regulatory limits applicable to organic fertilizers and soil amendments.

Metal	Law Limits (mg kg^−1^ d.s.)		PAPs (mg kg^−1^ d.s.)
	(Reg. EU 2019/1009) [33]	(Reg. EU 2021/1165) [35]	
Cadmium	1.5	0.7	≤0.01
Lead	120	45	4.3 ± 0.9
Chromium, total	-	70	1.86 ± 0.03
Chromium VI	2.0	Not detectable	-
Mercury	1.0	0.4	1.3 ± 0.1
Nickel	50	25	3.8 ± 0.2
Copper	300	70	24 ± 2
Zinc	800	200	50 ± 1
Inorganic Arsenic	40	-	3 ± 1

**Table 3:** Germination Index (GI, %) at different compound concentrations for *Lepidium sativum*. Data are expressed as mean ± SD of three independent Petri dishes (*n* = 3). The lowercase letters indicate significant differences among concentrations, according to one-way ANOVA followed by Tukey’s HSD multiple-comparison test (*p* < 0.05). Overall ANOVA: F-value = 341.38 *p* < 0.0001.

Case	Conc. % (*v*/*v*)	GI % Petri Dish 1	GI % Petri Dish 2	GI % Petri Dish 3	GI % (Mean ± SD)	Tukey
I	1	90	98	97	95 ± 4	a
II	5	88	94	94	92 ± 4	a
III	10	88	94	93	92 ± 3	a
IV	15	80	94	86	87 ± 7	a
V	30	71	77	74	74 ± 3	b
VI	50	18	16	10	15 ± 4	c
VII	75	10	9	10	9.7 ± 0.6	cd
VIII	90	0	0	0	0.0 ± 0.0	d

As can be observed in Table 3, cases I–IV do not differ significantly, as well as VI and VII, while there is a significant difference among the groups V, VI and VIII, so the concentration of PAPs clearly influences the GI of *Lepidium sativum*.

**Table 4:** Germination Index (GI, %) at different compound concentrations for *Lactuca sativa.* Data are expressed as mean ± SD of three independent Petri dishes (*n* = 3). The lowercase letters indicate significant differences among concentrations according to one-way ANOVA followed by Tukey’s HSD multiple-comparison test (*p* < 0.05). Overall ANOVA: *F*_12,26_ = 576.47 *p* < 0.001.

Case	Conc. % (*v*/*v*)	GI % Petri Dish 1	GI % Petri Dish 2	GI % Petri Dish 3	GI % (Mean ± SD)	Tukey
I	0	100	100	100	100 ± 0	b
II	0.1	105	106	105	105.3 ± 0.6	b
III	0.5	106	104	104	105 ± 1	b
IV	1	150	137	149	145 ± 7	a
V	2	133	132	155	140 ± 13	a
VI	3	8	15	7	10 ± 4	c
VII	10	8	10	6	8 ± 2	c
VIII	20	2	3	2.5	2.5 ± 0.5	c
IX	30	1	0	0	0.3 ± 0.6	c
X	50	0	0	0	0	c
XI	75	0	0	0	0	c
XII	90	0	0	0	0	c
XIII	100	0	0	0	0	c

As can be observed for Lactuca sativa, cases I–III do not differ significantly, as well as IV and V and VI–XIII, while there is a significant difference among I–IV and V–VI. As observed for Lepidium sativum, also in this case, the concentration of PAPs clearly influences the GI.

**Table 5:** Trials conducted with *Lactuca sativa* and *Lepidium sativum* (**C** commercial nutrient solution, total hydroponic tank volume 3000 mL).

Plant	C Content	PAPs Content g L^−1^	Growth *
*Lactuca sativa*	30 mL	-	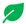 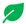 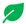
	30 mL	0.75	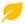
	30 mL	1.50	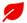
	-	0.75	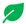 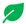
	-	1.50	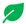
	-	3.00	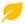
	-	4.50	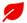
*Lepidium sativum*	30 mL	-	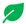 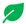 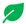
	30 mL	0.75	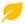
	-	0.75	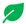 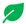
	-	1.50	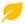
	-	3.00	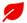

* 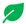

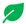

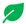
 Best-level growth, like that of the control; 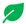

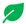
 very good growth; 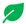 good growth; 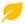 slightly reduced growth compared to the control; 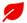 strongly reduced growth compared to the control.

**Table 6:** Length of the plant at different compound concentrations for *Lepidium sativum*. Data are expressed as mean ± SD of four independent pots (*n* = 4). The lowercase letters indicate significant differences among concentrations, according to one-way ANOVA followed by Tukey’s HSD multiple-comparison test (*p* < 0.05). Overall ANOVA: F-value = 31.81, *p* < 0.001.

Pot System	Mean Plant Length ± SD (cm)	Tukey
C (only peat moss)	4.5 ± 0.8	cd
PAPs 1 g kg^−1^	7 ± 1	ab
PAPs 5 g kg^−1^	7.8 ± 0.0	a
PAPs 10 g kg^−1^	6 ± 1	bc
PAPs 20 g kg^−1^	3.3 ± 0.3	de
PAPs 30 g kg^−1^	2.1 ± 0.7	e

## Data Availability

Data are contained within the article.

## Supplementary Materials

The following supporting information can be downloaded at: https://www.mdpi.com/article/10.3390/plants15203120/s1, Figure S1: Some Petri dishes of germination tests on *Lepidium sativum*. Section S1: ANOVA Results of Germination index for *Lepidium sativum*; Section S2: ANOVA Results of Germination index for *Lactuca sativa*; Section S3: ANOVA Results of Hydroponic test for *Lactuca sativa*; Section S4: ANOVA Results of Hydroponic test for *Lepidium sativum*; Section S5: ANOVA Results of Pot test for *Lepidium sativum*; Table S1. Analytical parameters of quantitative analysis (ICP-OES).
