# Supplementary material for: Agronomic Performance and Elemental Uptake of a Potential Fertilizer Derived from Microwave-Treated Animal By-Products in Lepidium sativum and Lactuca sativa
Source: Plants (Basel). 2026 Oct 12;15(20):3120. doi: 10.3390/plants15203120 (PMC13708627; doi:10.3390/plants15203120)
Supplement: Supplementary file 1 [file plants-15-03120-s001.zip › plants-4595040-supplementary.pdf]

Agronomic Performance and Elemental Uptake of a Potential Fertilizer Derived from Microwave-Treated Animal By-Products in *Lepidium sativum* and *Lactuca sativa*

Alba Cao [1], Costantino Palmas [2], Giulia Urracci [3], Marco Dettori [3], Tiziana Pivetta [1,*]

[1] Chemical and Geological Sciences Department, University of Cagliari, Italy
[2] Scientific Coordinator of the AGRIS–Caritas Protocol, Caritas Diocesana di Cagliari, Cagliari, Italy
[3] Agenzia AGRIS Sardegna, Viale Trieste 111, 09123 Cagliari, Italy

* Corresponding authors: Tiziana Pivetta e-mail: tpivetta@unica.it

Supplementary materials

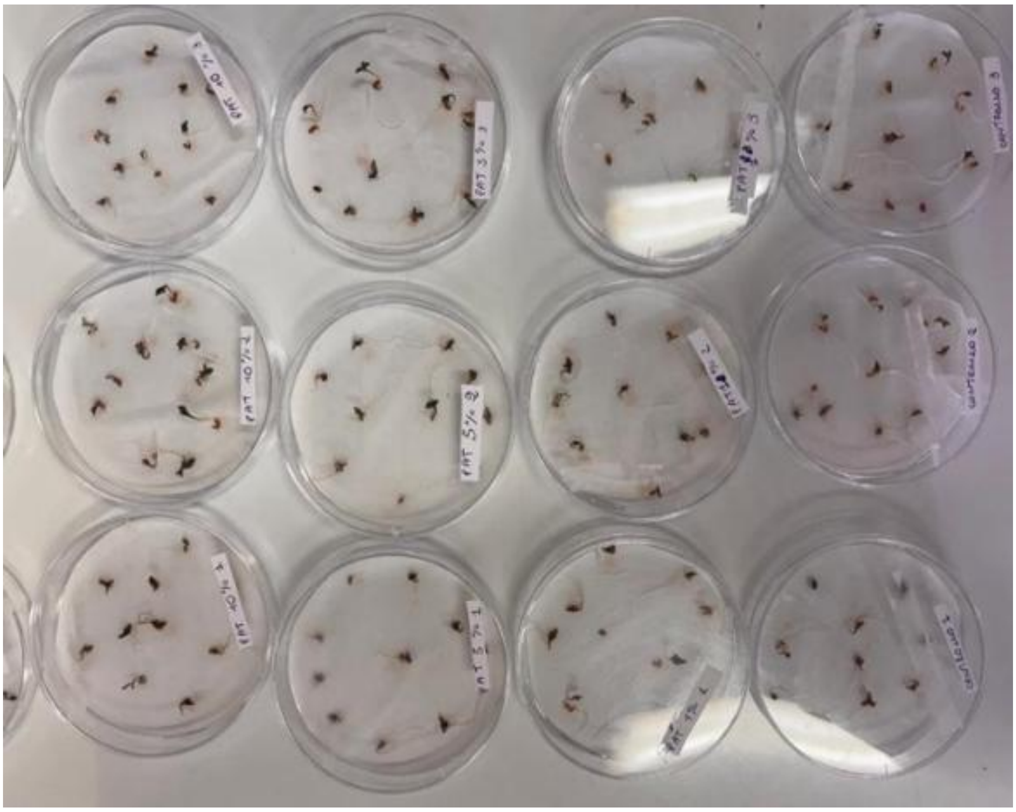

Figure S1. Some Petri dishes of germination tests on *Lepidium sativum*.

Section S1: ANOVA Results of Germination index for *Lepidium sativum*

Descriptive Statistics

	N Analysis	N Missing	Mean	Standard Deviation	SE of Mean
0.01	1	0	90	--	--
0.05	3	0	92	3.4641	2
0.1	3	0	91.66667	3.21455	1.85592
0.15	3	0	86.66667	7.02377	4.05518
0.3	3	0	74	3	1.73205
0.5	3	0	14.66667	4.16333	2.4037
0.75	3	0	9.66667	0.57735	0.33333
0.9	3	0	0	0	0

ANOVA

Overall ANOVA

	DF	Sum of Squares	Mean Square	F Value	Prob>F
Model	7	33568.42424	4795.48918	341.37381	<0.0001
Error	14	196.66667	14.04762		
Total	21	33765.09091			

Null Hypothesis: The means of all levels are equal.
Alternative Hypothesis: The means of one or more levels are different.
At the 0.05 level, the population means are significantly different.

Fit Statistics

R-Square	Coeff Var	Root MSE	Data Mean
0.99418	0.06894	3.74802	54.36364

Means Comparisons

Tukey Test

	MeanDiff	SEM	q Value	Prob	Alpha	Sig	LCL	UCL
0.05 0.01	2	4.32784	0.65354	0.99968	0.05	0	-13.2715	17.2715
0.1 0.01	1.66667	4.32784	0.54462	0.9999	0.05	0	-13.60484	16.93817
0.1 0.05	-0.33333	3.06024	0.15404	1	0.05	0	-11.13192	10.46525
0.15 0.01	-3.33333	4.32784	1.08924	0.99221	0.05	0	-18.60484	11.93817
0.15 0.05	-5.33333	3.06024	2.46467	0.66314	0.05	0	-16.13192	5.46525
0.15 0.1	-5	3.06024	2.31062	0.72492	0.05	0	-15.79858	5.79858
0.3 0.01	-16	4.32784	5.22835	0.03709	0.05	1	-31.2715	-0.7285
0.3 0.05	-18	3.06024	8.31825	7.79858E-4	0.05	1	-28.79858	-7.20142
0.3 0.1	-17.66667	3.06024	8.1642	9.37344E-4	0.05	1	-28.46525	-6.86808
0.3 0.15	-12.66667	3.06024	5.85358	0.01676	0.05	1	-23.46525	-1.86808
0.5 0.01	-75.33333	4.32784	24.61679	<0.0001	0.05	1	-90.60484	-60.06183
0.5 0.05	-77.33333	3.06024	35.73765	<0.0001	0.05	1	-88.13192	-66.53475
0.5 0.1	-77	3.06024	35.58361	<0.0001	0.05	1	-87.79858	-66.20142
0.5 0.15	-72	3.06024	33.27298	<0.0001	0.05	1	-82.79858	-61.20142
0.5 0.3	-59.33333	3.06024	27.4194	<0.0001	0.05	1	-70.13192	-48.53475
0.75 0.01	-80.33333	4.32784	26.25065	<0.0001	0.05	1	-95.60484	-65.06183
0.75 0.05	-82.33333	3.06024	38.04827	<0.0001	0.05	1	-93.13192	-71.53475
0.75 0.1	-82	3.06024	37.89423	<0.0001	0.05	1	-92.79858	-71.20142
0.75 0.15	-77	3.06024	35.58361	<0.0001	0.05	1	-87.79858	-66.20142
0.75 0.3	-64.33333	3.06024	29.73003	<0.0001	0.05	1	-75.13192	-53.53475
0.75 0.5	-5	3.06024	2.31062	0.72492	0.05	0	-15.79858	5.79858
0.9 0.01	-90	4.32784	29.40944	<0.0001	0.05	1	-105.2715	-74.7285
0.9 0.05	-92	3.06024	42.51548	<0.0001	0.05	1	-102.79858	-81.20142
0.9 0.1	-91.66667	3.06024	42.36144	<0.0001	0.05	1	-102.46525	-80.86808
0.9 0.15	-86.66667	3.06024	40.05082	<0.0001	0.05	1	-97.46525	-75.86808
0.9 0.3	-74	3.06024	34.19723	<0.0001	0.05	1	-84.79858	-63.20142
0.9 0.5	-14.66667	3.06024	6.77783	0.00517	0.05	1	-25.46525	-3.86808
0.9 0.75	-9.66667	3.06024	4.46721	0.09497	0.05	0	-20.46525	1.13192

Grouping Letters Table

Tukey Test

	Mean	Groups			
0.05	92	A			
0.1	91.66667	A			
0.01	90	A			
0.15	86.66667	A			
0.3	74		B		
0.5	14.66667			C	
0.75	9.66667			C	D
0.9	0				D

Means that do not share a letter are significantly different.

Sig equals 1 indicates that the difference of the means is significant at the 0.05 level.
Sig equals 0 indicates that the difference of the means is not significant at the 0.05 level.

Homogeneity of Variance Test

Levene's Test(Absolute Deviations)

	DF	Sum of Squares	Mean Square	F Value	Prob>F
Model	7	53.60269	7.65753	2.43239	0.07436
Error	14	44.07407	3.14815		

At the 0.05 level, the population variances are not significantly different.

Powers

	Alpha	Sample Size	Power
Actual Power	0.05	22	1

The sample size is the total size for all groups.

Summary

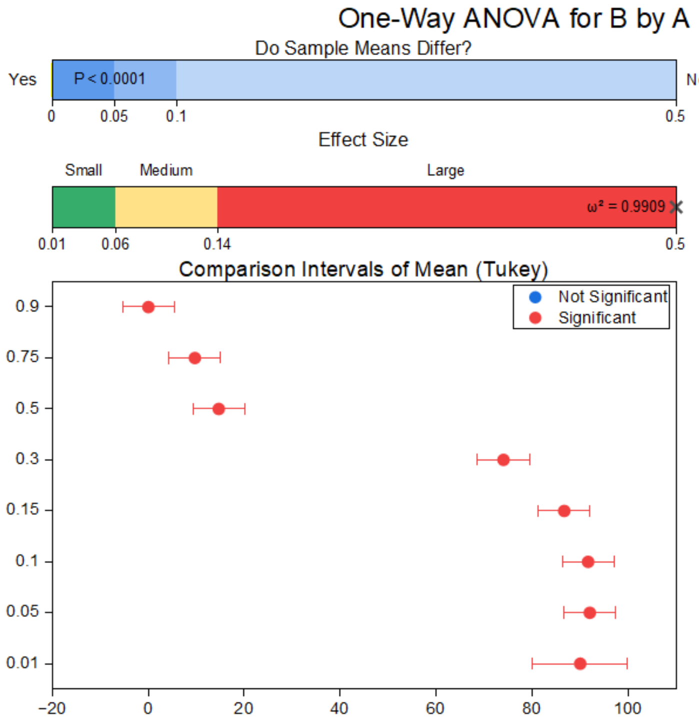

Which means differ?

#	Sample	Differs From
1	0.9	3 4 5 6 7 8
2	0.75	4 5 6 7 8
3	0.5	1 4 5 6 7 8
4	0.3	1 2 3 5 6 7 8
5	0.15	1 2 3 4
6	0.1	1 2 3 4
7	0.05	1 2 3 4
8	0.01	1 2 3 4

Statistics

Sample	Mean	SD	95% CI of Mean
0.9	0	0	(0.000, 0.000)
0.75	9.667	0.577	(8.232, 11.101)
0.5	14.667	4.163	(4.324, 25.009)
0.3	74	3	(66.548, 81.452)
0.15	86.667	7.024	(69.219, 104.115)
0.1	91.667	3.215	(83.681, 99.652)
0.05	92	3.464	(83.395, 100.605)
0.01	90	--	(--, --)

- At the 0.05 level, the population means are significantly different.
- Non-overlapping red intervals indicate differences between the means; consider their size for practical significance.

Diagnostic

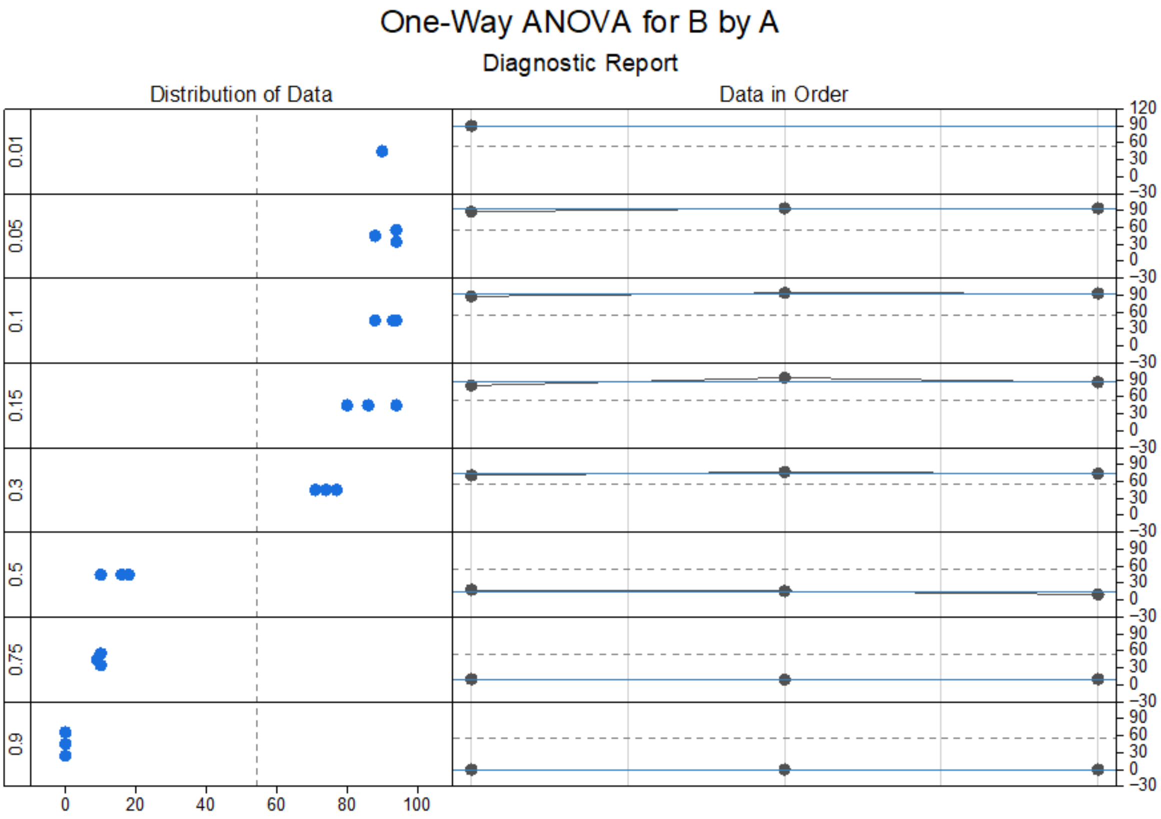

Box Charts

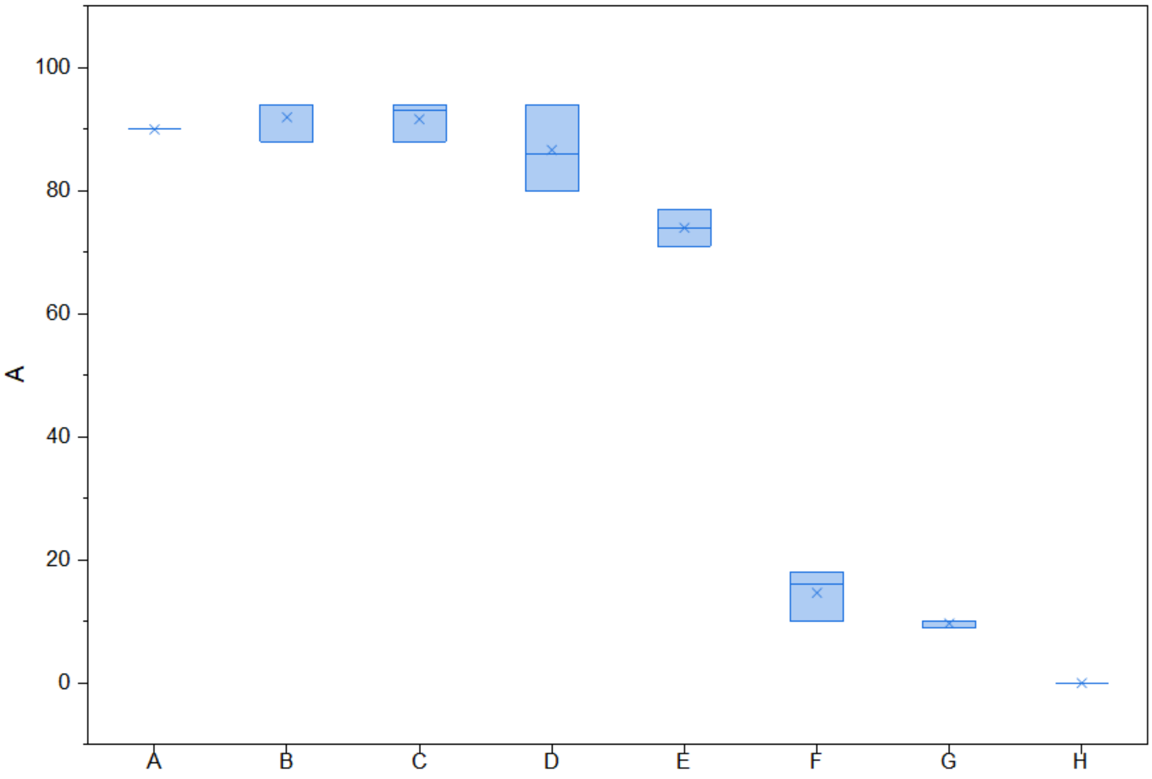

⊟ **Means Plot (SE as Error)**

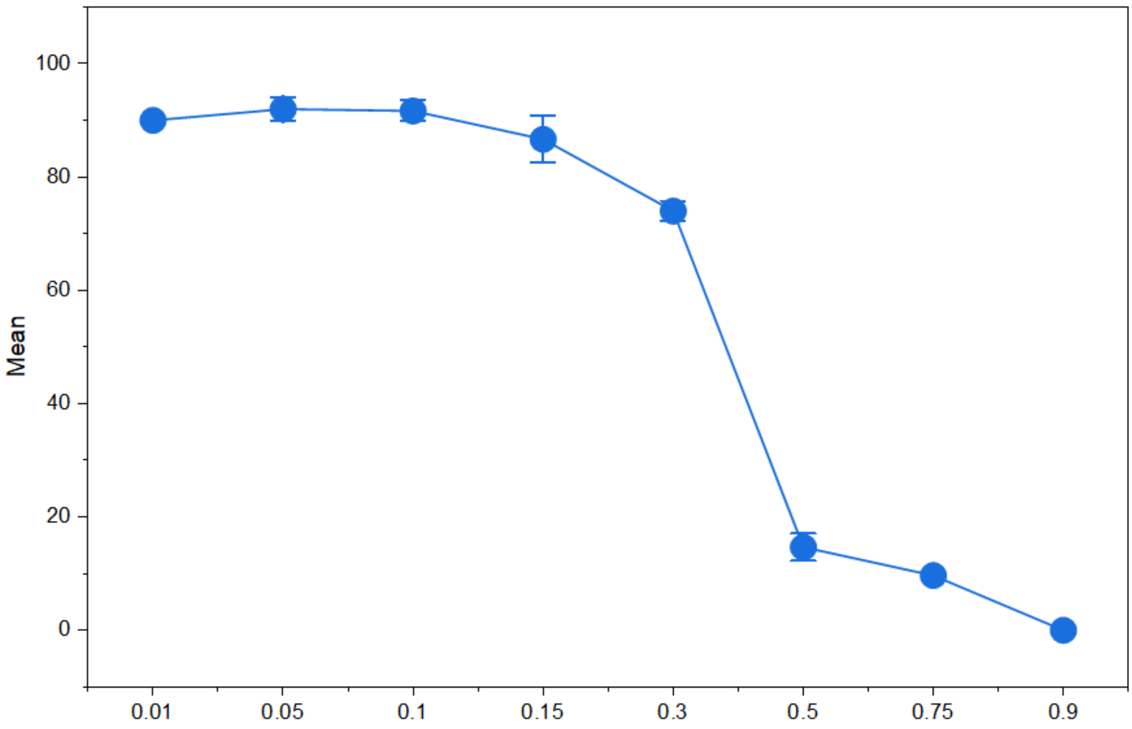

⊟ **Means Comparison Plot**

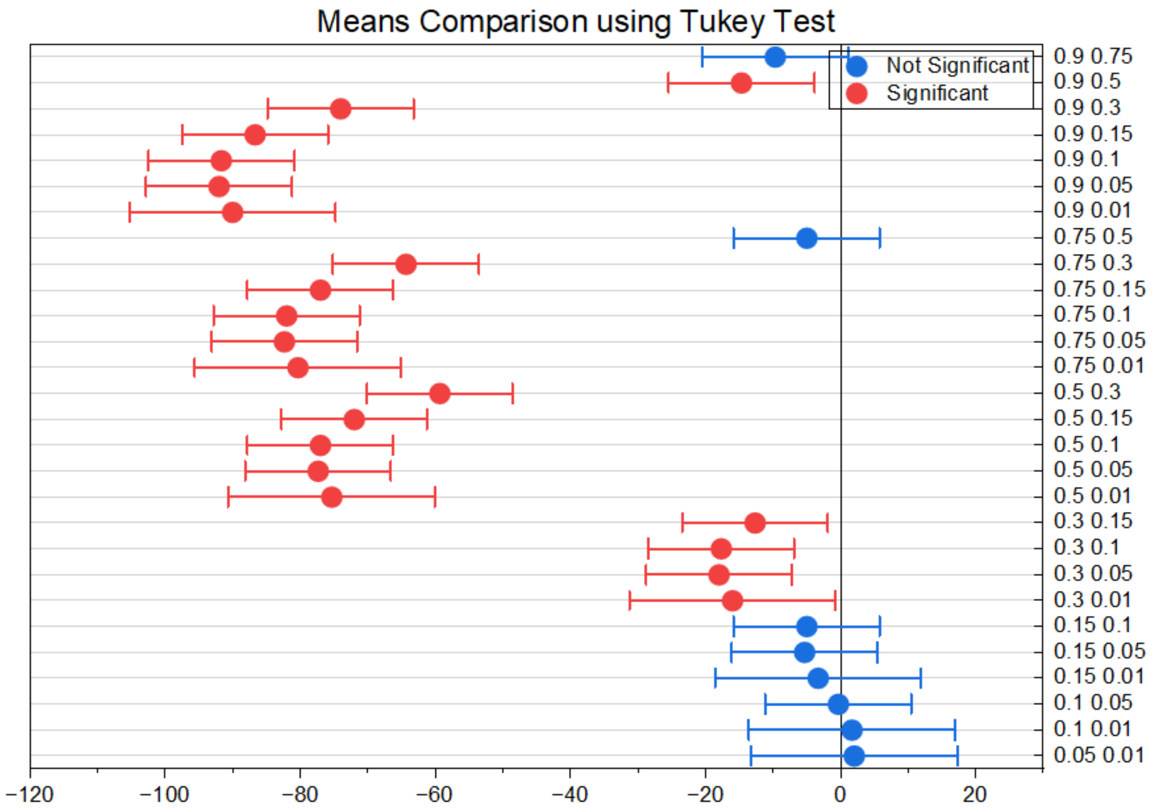

Power

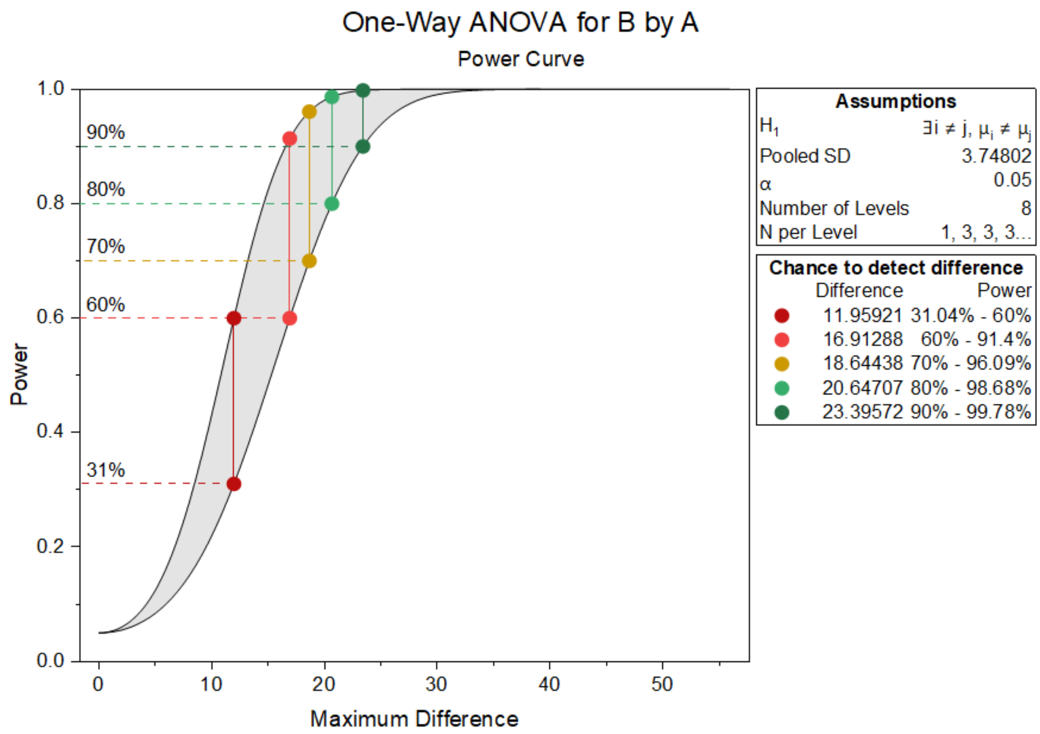

The power curve shows how the statistical power of the test changes as the difference in means varies. Power is the probability of rejecting the null hypothesis when the true difference differs from 0. A power of 80% is commonly considered sufficient. If the power at the expected difference is too low, consider increasing the sample size.

Section S2: ANOVA Results of Germination index for *Lactuca sativa*

Descriptive Statistics

	N Analysis	N Missing	Mean	Standard Deviation	SE of Mean
0	3	0	100	0	0
0.1	3	0	105.33333	0.57735	0.33333
0.5	3	0	104.66667	1.1547	0.66667
1	3	0	145.33333	7.23418	4.17665
2	3	0	140	13	7.50555
3	3	0	10	4.3589	2.51661
10	3	0	8	2	1.1547
20	3	0	2.5	0.5	0.28868
30	3	0	0.33333	0.57735	0.33333
50	3	0	0	0	0
75	3	0	0	0	0
90	3	0	0	0	0
100	3	0	0	0	0

ANOVA

Overall ANOVA

	DF	Sum of Squares	Mean Square	F Value	Prob>F
Model	12	131212.92308	10934.41026	576.46773	<0.0001
Error	26	493.16667	18.96795		
Total	38	131706.08974			

Null Hypothesis: The means of all levels are equal.
Alternative Hypothesis: The means of one or more levels are different.
At the 0.05 level, the population means are significantly different.

Fit Statistics

R-Square	Coeff Var	Root MSE	Data Mean
0.99626	0.09189	4.35522	47.39744

Means Comparisons

Tukey Test

	MeanDiff	SEM	q Value	Prob	Alpha	Sig	LCL	UCL
0.1 0	5.33333	3.55602	2.12104	0.94343	0.05	0	-7.5894	18.25607
0.5 0	4.66667	3.55602	1.85591	0.97819	0.05	0	-8.25607	17.5894
0.5 0.1	-0.66667	3.55602	0.26513	1	0.05	0	-13.5894	12.25607
1 0	45.33333	3.55602	18.02885	<0.0001	0.05	1	32.4106	58.25607
1 0.1	40	3.55602	15.90781	<0.0001	0.05	1	27.07727	52.92273
1 0.5	40.66667	3.55602	16.17294	<0.0001	0.05	1	27.74393	53.5894
2 0	40	3.55602	15.90781	<0.0001	0.05	1	27.07727	52.92273
2 0.1	34.66667	3.55602	13.78677	<0.0001	0.05	1	21.74393	47.5894
2 0.5	35.33333	3.55602	14.0519	<0.0001	0.05	1	22.4106	48.25607
2 1	-5.33333	3.55602	2.12104	0.94343	0.05	0	-18.25607	7.5894
3 0	-90	3.55602	35.79258	<0.0001	0.05	1	-102.92273	-77.07727
3 0.1	-95.33333	3.55602	37.91362	<0.0001	0.05	1	-108.25607	-82.4106
3 0.5	-94.66667	3.55602	37.64849	<0.0001	0.05	1	-107.5894	-81.74393
3 1	-135.33333	3.55602	53.82143	<0.0001	0.05	1	-148.25607	-122.4106
3 2	-130	3.55602	51.70039	<0.0001	0.05	1	-142.92273	-117.07727
10 0	-92	3.55602	36.58797	<0.0001	0.05	1	-104.92273	-79.07727
10 0.1	-97.33333	3.55602	38.70901	<0.0001	0.05	1	-110.25607	-84.4106
10 0.5	-96.66667	3.55602	38.44388	<0.0001	0.05	1	-109.5894	-83.74393
10 1	-137.33333	3.55602	54.61682	<0.0001	0.05	1	-150.25607	-124.4106
10 2	-132	3.55602	52.49578	<0.0001	0.05	1	-144.92273	-119.07727
10 3	-2	3.55602	0.79539	0.99999	0.05	0	-14.92273	10.92273
20 0	-97.5	3.55602	38.77529	<0.0001	0.05	1	-110.42273	-84.57727
20 0.1	-102.83333	3.55602	40.89633	<0.0001	0.05	1	-115.75607	-89.9106
20 0.5	-102.16667	3.55602	40.6312	<0.0001	0.05	1	-115.0894	-89.24393
20 1	-142.83333	3.55602	56.80414	<0.0001	0.05	1	-155.75607	-129.9106
20 2	-137.5	3.55602	54.6831	<0.0001	0.05	1	-150.42273	-124.57727
20 3	-7.5	3.55602	2.98271	0.65714	0.05	0	-20.42273	5.42273
20 10	-5.5	3.55602	2.18732	0.93083	0.05	0	-18.42273	7.42273
30 0	-99.66667	3.55602	39.63696	<0.0001	0.05	1	-112.5894	-86.74393
30 0.1	-105	3.55602	41.75801	<0.0001	0.05	1	-117.92273	-92.07727
30 0.5	-104.33333	3.55602	41.49287	<0.0001	0.05	1	-117.25607	-91.4106
30 1	-145	3.55602	57.66582	<0.0001	0.05	1	-157.92273	-132.07727
30 2	-139.66667	3.55602	55.54478	<0.0001	0.05	1	-152.5894	-126.74393
30 3	-9.66667	3.55602	3.84439	0.29764	0.05	0	-22.5894	3.25607
30 10	-7.66667	3.55602	3.049	0.62755	0.05	0	-20.5894	5.25607
30 20	-2.16667	3.55602	0.86167	0.99998	0.05	0	-15.0894	10.75607
50 0	-100	3.55602	39.76953	<0.0001	0.05	1	-112.92273	-87.07727
50 0.1	-105.33333	3.55602	41.89057	<0.0001	0.05	1	-118.25607	-92.4106
50 0.5	-104.66667	3.55602	41.62544	<0.0001	0.05	1	-117.5894	-91.74393
50 1	-145.33333	3.55602	57.79838	<0.0001	0.05	1	-158.25607	-132.4106
50 2	-140	3.55602	55.67734	<0.0001	0.05	1	-152.92273	-127.07727
50 3	-10	3.55602	3.97695	0.25487	0.05	0	-22.92273	2.92273
50 10	-8	3.55602	3.18156	0.56781	0.05	0	-20.92273	4.92273
50 20	-2.5	3.55602	0.99424	0.99993	0.05	0	-15.42273	10.42273
50 30	-0.33333	3.55602	0.13257	1	0.05	0	-13.25607	12.5894
75 0	-100	3.55602	39.76953	<0.0001	0.05	1	-112.92273	-87.07727
75 0.1	-105.33333	3.55602	41.89057	<0.0001	0.05	1	-118.25607	-92.4106
75 0.5	-104.66667	3.55602	41.62544	<0.0001	0.05	1	-117.5894	-91.74393
75 1	-145.33333	3.55602	57.79838	<0.0001	0.05	1	-158.25607	-132.4106
75 2	-140	3.55602	55.67734	<0.0001	0.05	1	-152.92273	-127.07727
75 3	-10	3.55602	3.97695	0.25487	0.05	0	-22.92273	2.92273
75 10	-8	3.55602	3.18156	0.56781	0.05	0	-20.92273	4.92273
75 20	-2.5	3.55602	0.99424	0.99993	0.05	0	-15.42273	10.42273

50 30	-0.33333	3.55602	0.13257	1	0.05	0	-13.25607	12.5894
75 0	-100	3.55602	39.76953	<0.0001	0.05	1	-112.92273	-87.07727
75 0.1	-105.33333	3.55602	41.89057	<0.0001	0.05	1	-118.25607	-92.4106
75 0.5	-104.66667	3.55602	41.62544	<0.0001	0.05	1	-117.5894	-91.74393
75 1	-145.33333	3.55602	57.79838	<0.0001	0.05	1	-158.25607	-132.4106
75 2	-140	3.55602	55.67734	<0.0001	0.05	1	-152.92273	-127.07727
75 3	-10	3.55602	3.97695	0.25487	0.05	0	-22.92273	2.92273
75 10	-8	3.55602	3.18156	0.56781	0.05	0	-20.92273	4.92273
75 20	-2.5	3.55602	0.99424	0.99993	0.05	0	-15.42273	10.42273
75 30	-0.33333	3.55602	0.13257	1	0.05	0	-13.25607	12.5894
75 50	0	3.55602	0	1	0.05	0	-12.92273	12.92273
90 0	-100	3.55602	39.76953	<0.0001	0.05	1	-112.92273	-87.07727
90 0.1	-105.33333	3.55602	41.89057	<0.0001	0.05	1	-118.25607	-92.4106
90 0.5	-104.66667	3.55602	41.62544	<0.0001	0.05	1	-117.5894	-91.74393
90 1	-145.33333	3.55602	57.79838	<0.0001	0.05	1	-158.25607	-132.4106
90 2	-140	3.55602	55.67734	<0.0001	0.05	1	-152.92273	-127.07727
90 3	-10	3.55602	3.97695	0.25487	0.05	0	-22.92273	2.92273
90 10	-8	3.55602	3.18156	0.56781	0.05	0	-20.92273	4.92273
90 20	-2.5	3.55602	0.99424	0.99993	0.05	0	-15.42273	10.42273
90 30	-0.33333	3.55602	0.13257	1	0.05	0	-13.25607	12.5894
90 50	0	3.55602	0	1	0.05	0	-12.92273	12.92273
90 75	0	3.55602	0	1	0.05	0	-12.92273	12.92273
100 0	-100	3.55602	39.76953	<0.0001	0.05	1	-112.92273	-87.07727
100 0.1	-105.33333	3.55602	41.89057	<0.0001	0.05	1	-118.25607	-92.4106
100 0.5	-104.66667	3.55602	41.62544	<0.0001	0.05	1	-117.5894	-91.74393
100 1	-145.33333	3.55602	57.79838	<0.0001	0.05	1	-158.25607	-132.4106
100 2	-140	3.55602	55.67734	<0.0001	0.05	1	-152.92273	-127.07727
100 3	-10	3.55602	3.97695	0.25487	0.05	0	-22.92273	2.92273
100 10	-8	3.55602	3.18156	0.56781	0.05	0	-20.92273	4.92273
100 20	-2.5	3.55602	0.99424	0.99993	0.05	0	-15.42273	10.42273
100 30	-0.33333	3.55602	0.13257	1	0.05	0	-13.25607	12.5894
100 50	0	3.55602	0	1	0.05	0	-12.92273	12.92273
100 75	0	3.55602	0	1	0.05	0	-12.92273	12.92273
100 90	0	3.55602	0	1	0.05	0	-12.92273	12.92273

Grouping Letters Table

Tukey Test

	Mean	Groups		
1	145.33333	A		
2	140	A		
0.1	105.33333		B	
0.5	104.66667		B	
0	100		B	
3	10			C
10	8			C
20	2.5			C
30	0.33333			C
50	0			C
75	0			C
90	0			C
100	0			C

Means that do not share a letter are significantly different.

Sig equals 1 indicates that the difference of the means is significant at the 0.05 level.
Sig equals 0 indicates that the difference of the means is not significant at the 0.05 level.

Homogeneity of Variance Test

Levene's Test(Absolute Deviations)

	DF	Sum of Squares	Mean Square	F Value	Prob>F
Model	12	320.04558	26.67047	11.95191	<0.0001
Error	26	58.01852	2.23148		

At the 0.05 level, the population variances are significantly different.

Powers

	Alpha	Sample Size	Power
Actual Power	0.05	39	1

The sample size is the total size for all groups.

One-Way ANOVA for B by A

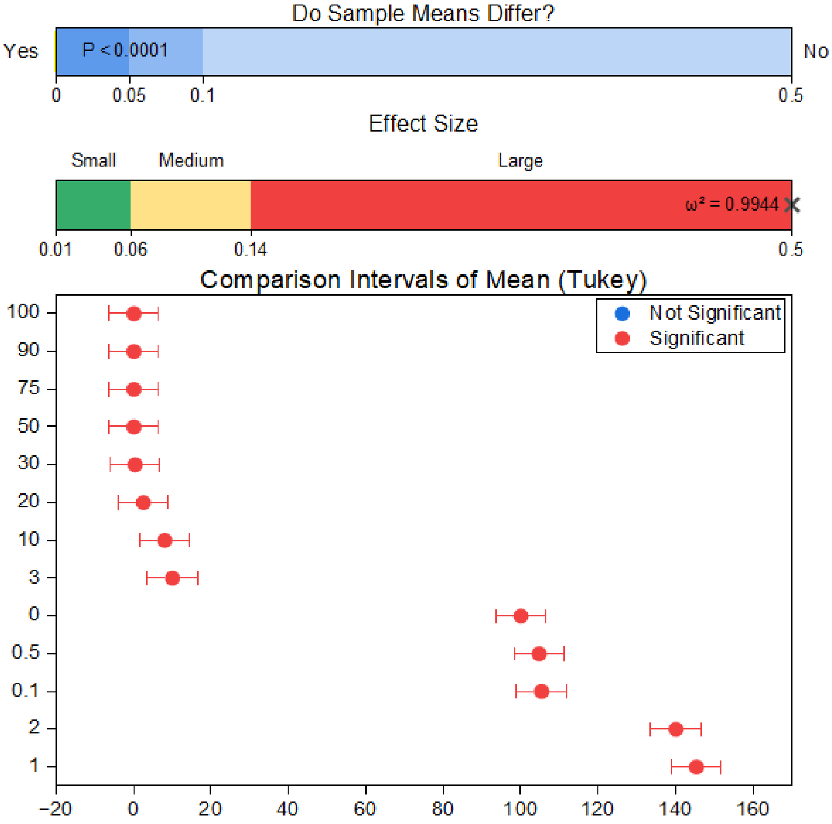

Which means differ?

#	Sample	Differs From
1	100	9 10 11 12 13
2	90	9 10 11 12 13
3	75	9 10 11 12 13
4	50	9 10 11 12 13
5	30	9 10 11 12 13
6	20	9 10 11 12 13
7	10	9 10 11 12 13
8	3	9 10 11 12 13
9	0	1 2 3 4 5 6 7 8 12 13
10	0.5	1 2 3 4 5 6 7 8 12 13
11	0.1	1 2 3 4 5 6 7 8 12 13
12	2	1 2 3 4 5 6 7 8 9 10 11
13	1	1 2 3 4 5 6 7 8 9 10 11

Statistics

Sample	Mean	SD	95% CI of Mean
100	0	0	(0.000, 0.000)
90	0	0	(0.000, 0.000)
75	0	0	(0.000, 0.000)
50	0	0	(0.000, 0.000)
30	0.333	0.577	(-1.101, 1.768)
20	2.5	0.5	(1.258, 3.742)
10	8	2	(3.032, 12.968)
3	10	4.359	(-0.828, 20.828)
0	100	0	(100.000, 100.000)
0.5	104.667	1.155	(101.798, 107.535)
0.1	105.333	0.577	(103.899, 106.768)
2	140	13	(107.706, 172.294)
1	145.333	7.234	(127.363, 163.304)

- At the 0.05 level, the population means are significantly different.
- Non-overlapping red intervals indicate differences between the means; consider their size for practical significance.

⊟ **Means Plot (SE as Error)**

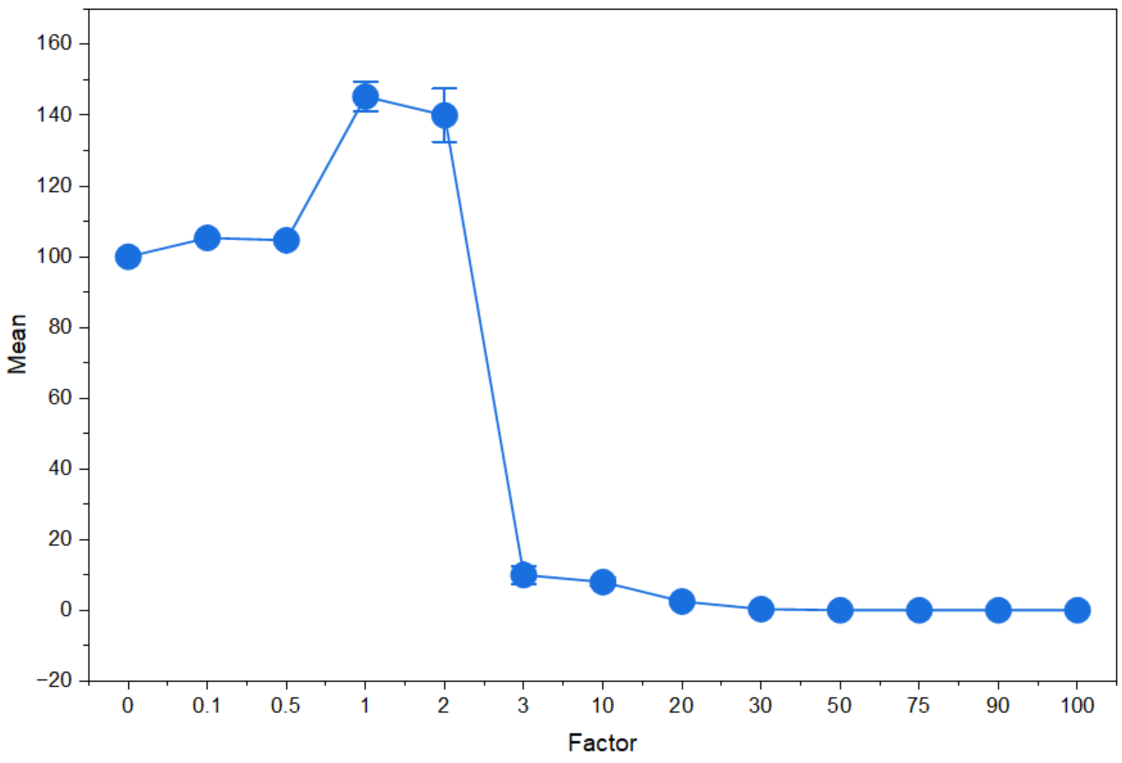

⊟ **Means Comparison Plot**

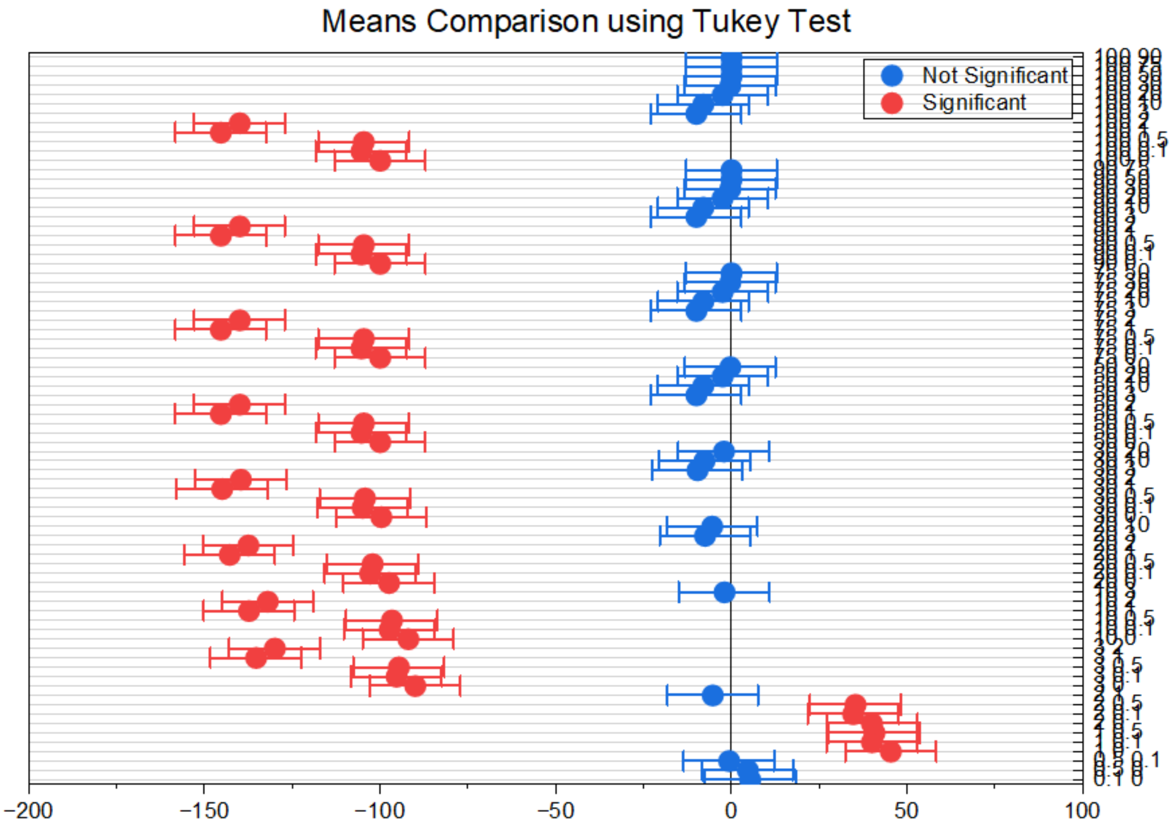

⊟ **Power**

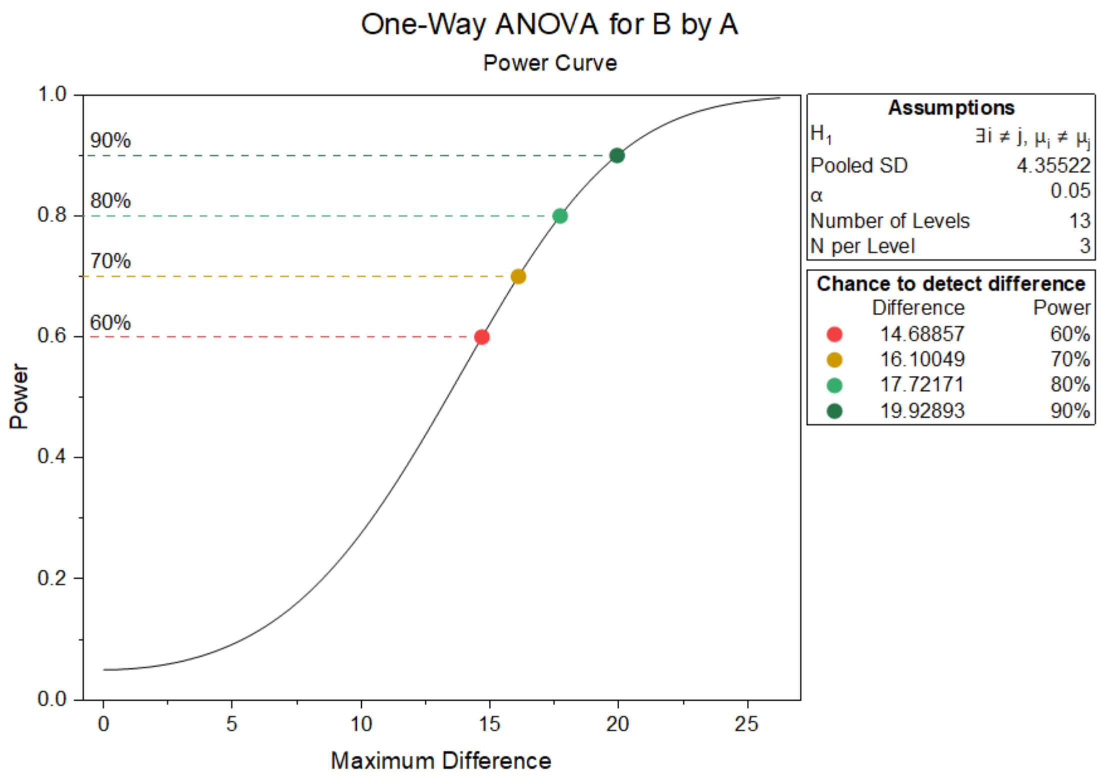

The power curve shows how the statistical power of the test changes as the difference in means varies. Power is the probability of rejecting the null hypothesis when the true difference differs from 0. A power of 80% is commonly considered sufficient. If the power at the expected difference is too low, consider increasing the sample size.

⊟ **Diagnostic**

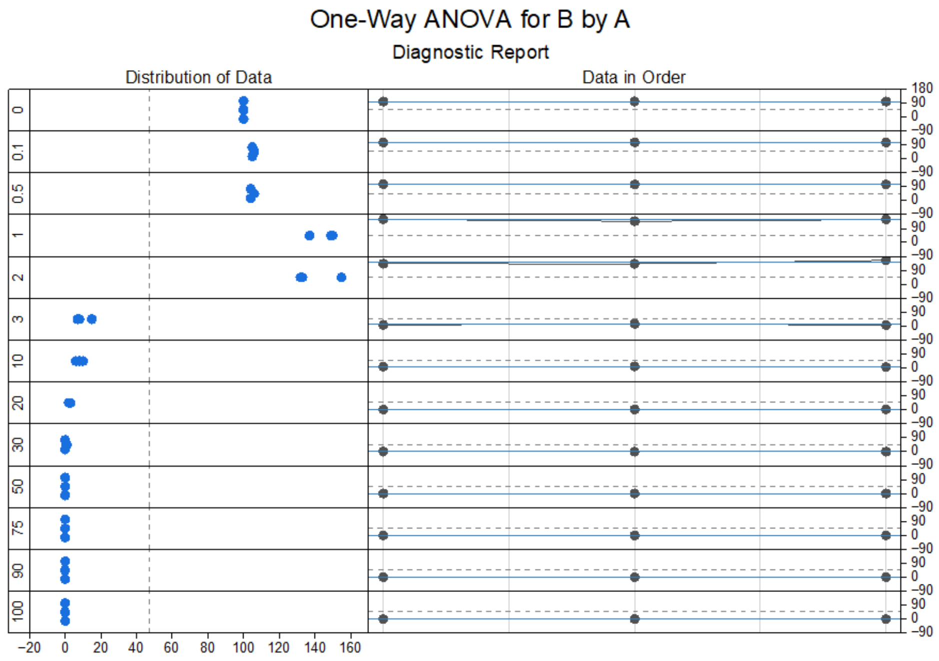

Box Charts

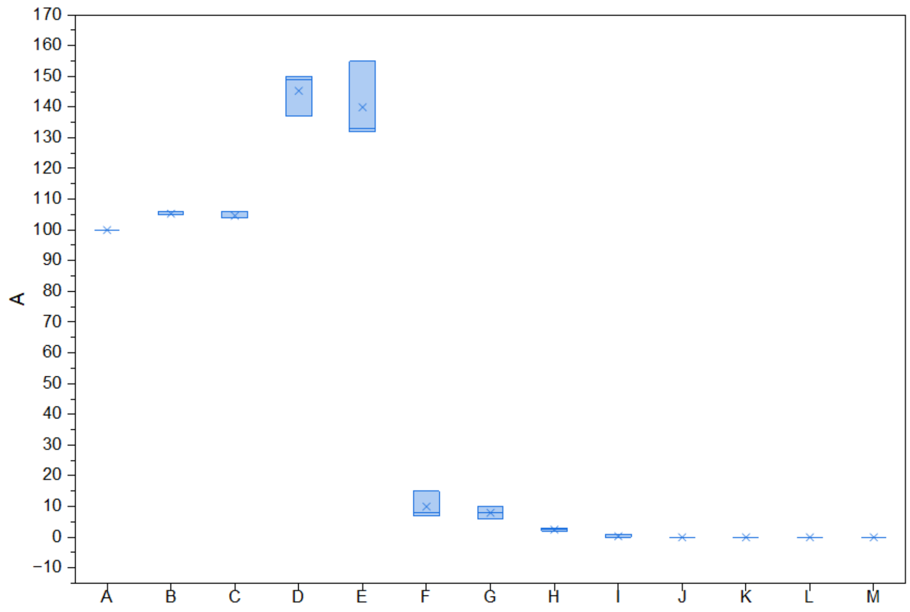

Section S3: ANOVA Results of Hydroponic test for *Lactuca sativa*

Descriptive Statistics

	N Analysis	N Missing	Mean	Standard Deviation	SE of Mean
C	2	0	10.9	0.56569	0.4
PAPs0.75	2	0	6.8	2.82843	2
PAPs1.50	2	0	3.5	2.12132	1.5

ANOVA

Overall ANOVA

	DF	Sum of Squares	Mean Square	F Value	Prob>F
Model	2	54.97333	27.48667	6.43214	0.08223
Error	3	12.82	4.27333		
Total	5	67.79333			

Null Hypothesis: The means of all levels are equal.
Alternative Hypothesis: The means of one or more levels are different.
At the 0.05 level, the population means are not significantly different.

Fit Statistics

R-Square	Coeff Var	Root MSE	Data Mean
0.8109	0.29253	2.0672	7.06667

Means Comparisons

Tukey Test

	MeanDiff	SEM	q Value	Prob	Alpha	Sig	LCL	UCL
PAPs0.75 C	-4.1	2.0672	2.80489	0.26269	0.05	0	-12.73825	4.53825
PAPs1.50 C	-7.4	2.0672	5.06248	0.07397	0.05	0	-16.03825	1.23825
PAPs1.50 PAPs0.75	-3.3	2.0672	2.25759	0.37225	0.05	0	-11.93825	5.33825

Grouping Letters Table

Tukey Test

	Mean	Groups
C	10.9	A
PAPs0.75	6.8	A
PAPs1.50	3.5	A

Means that do not share a letter are significantly different.

Sig equals 1 indicates that the difference of the means is significant at the 0.05 level.
Sig equals 0 indicates that the difference of the means is not significant at the 0.05 level.

Homogeneity of Variance Test

Levene's Test(Absolute Deviations)

	DF	Sum of Squares	Mean Square	F Value	Prob>F
Model	2	2.68	1.34	1.01919E31	<0.0001
Error	3	3.9443E-31	1.31477E-31		

At the 0.05 level, the population variances are significantly different.

Powers

	Alpha	Sample Size	Power
Actual Power	0.05	6	0.46163

The sample size is the total size for all groups.

Summary

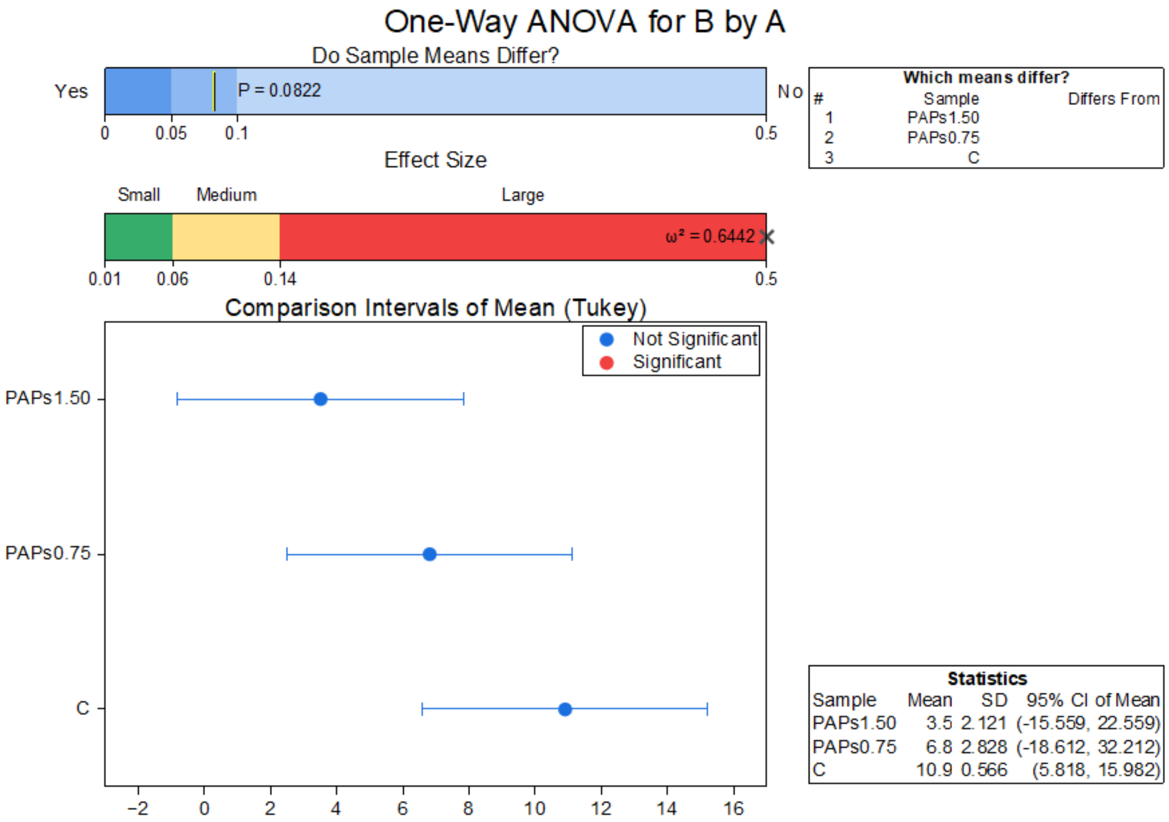

• At the 0.05 level, the population means are not significantly different.
• Blue intervals indicate no detected differences between the means.

⊟ Diagnostic

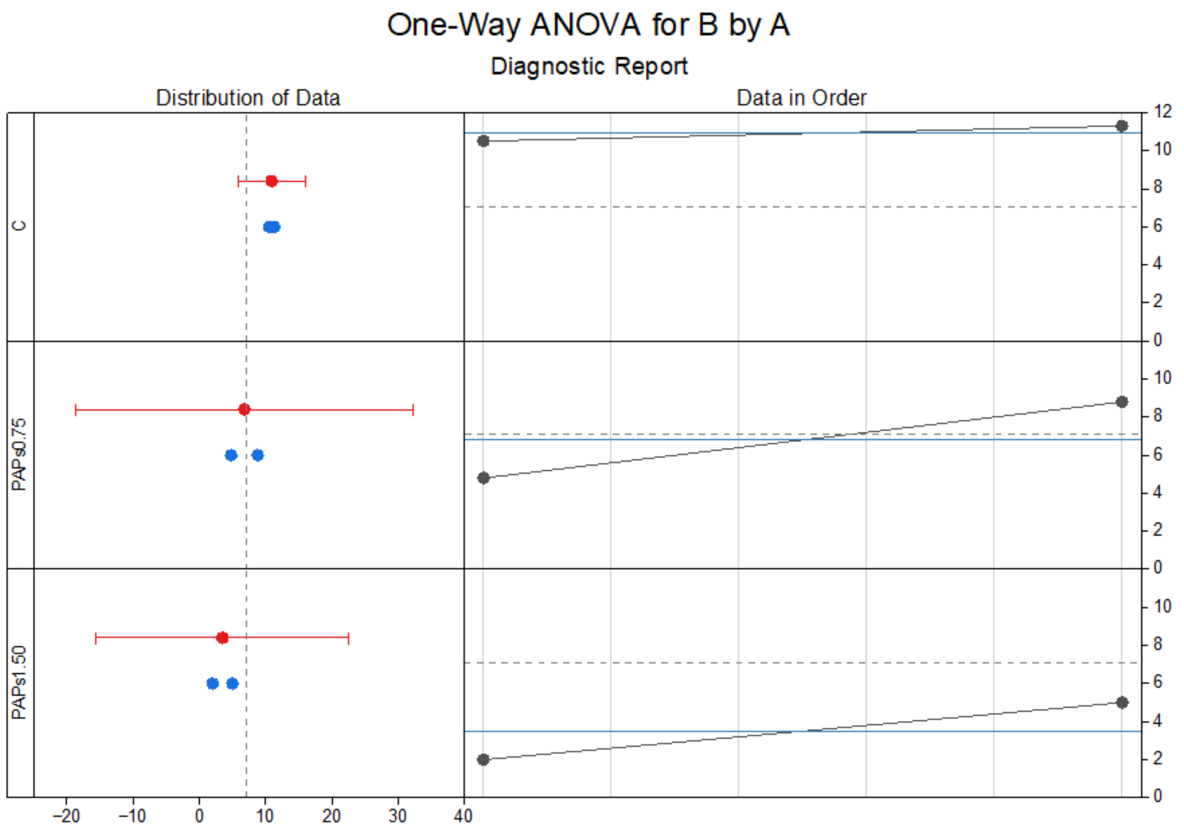

⊟ Box Charts

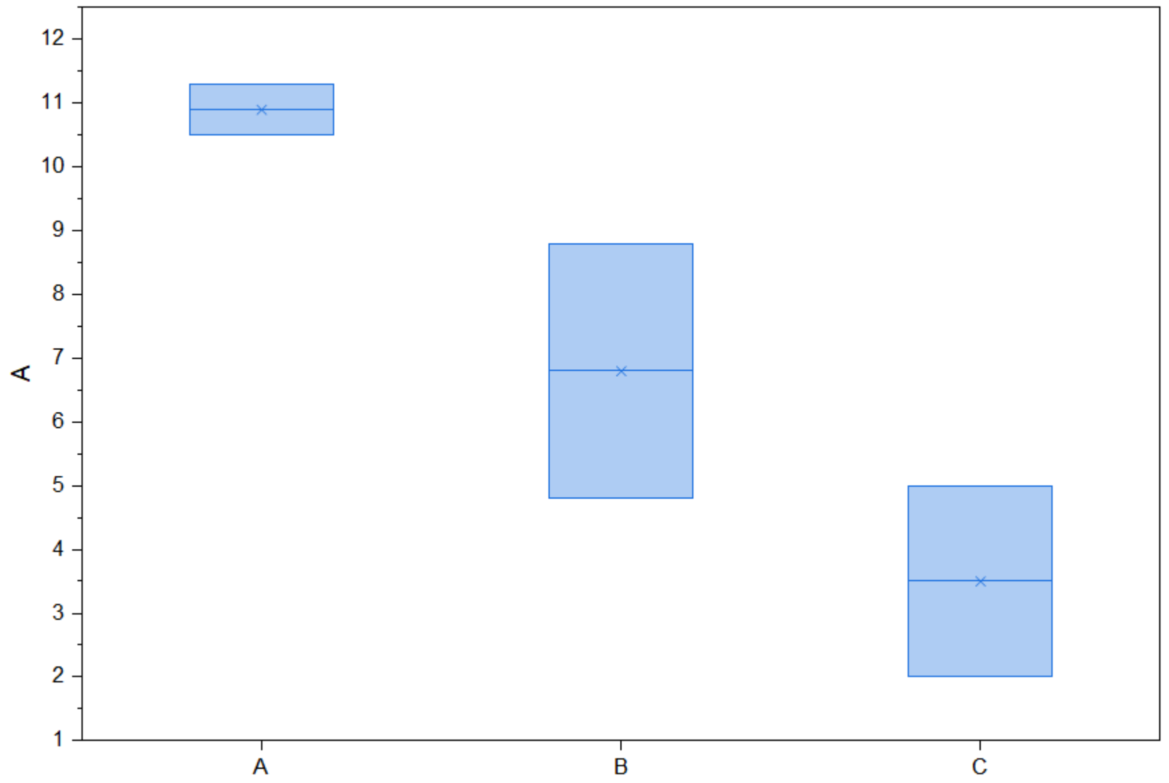

Means Plot (SE as Error)

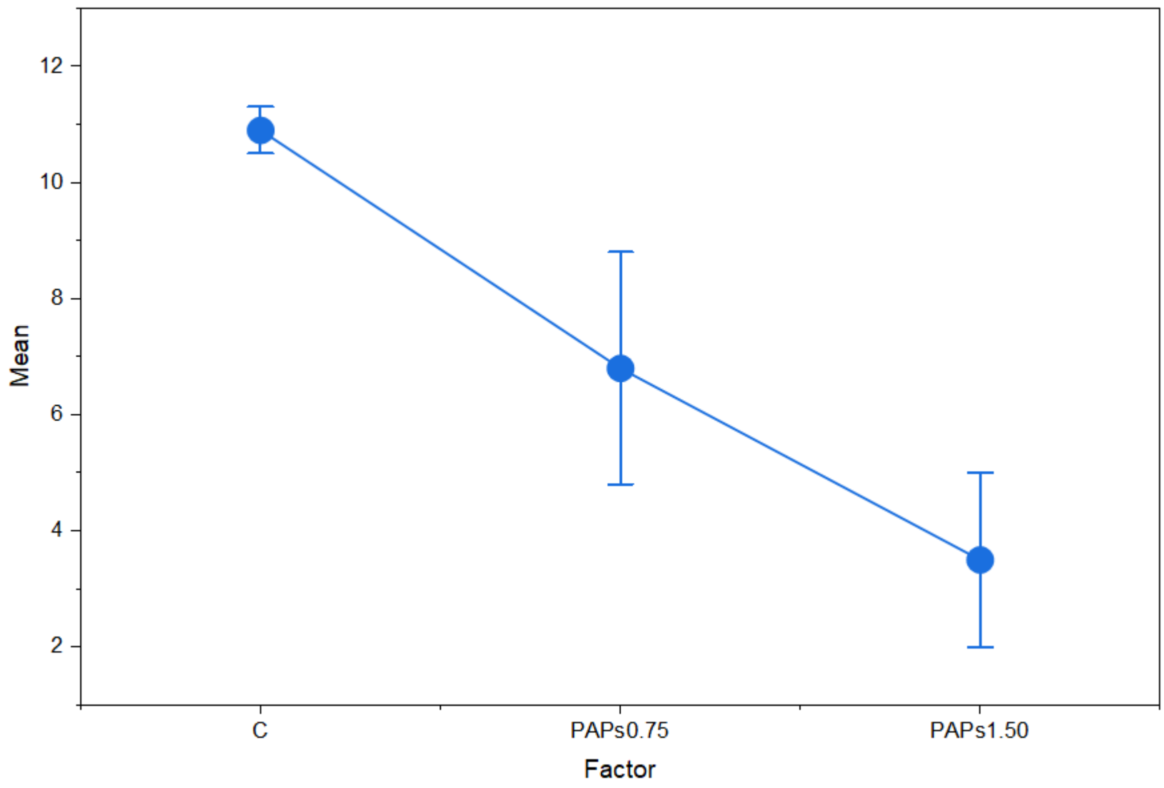

Means Comparison Plot

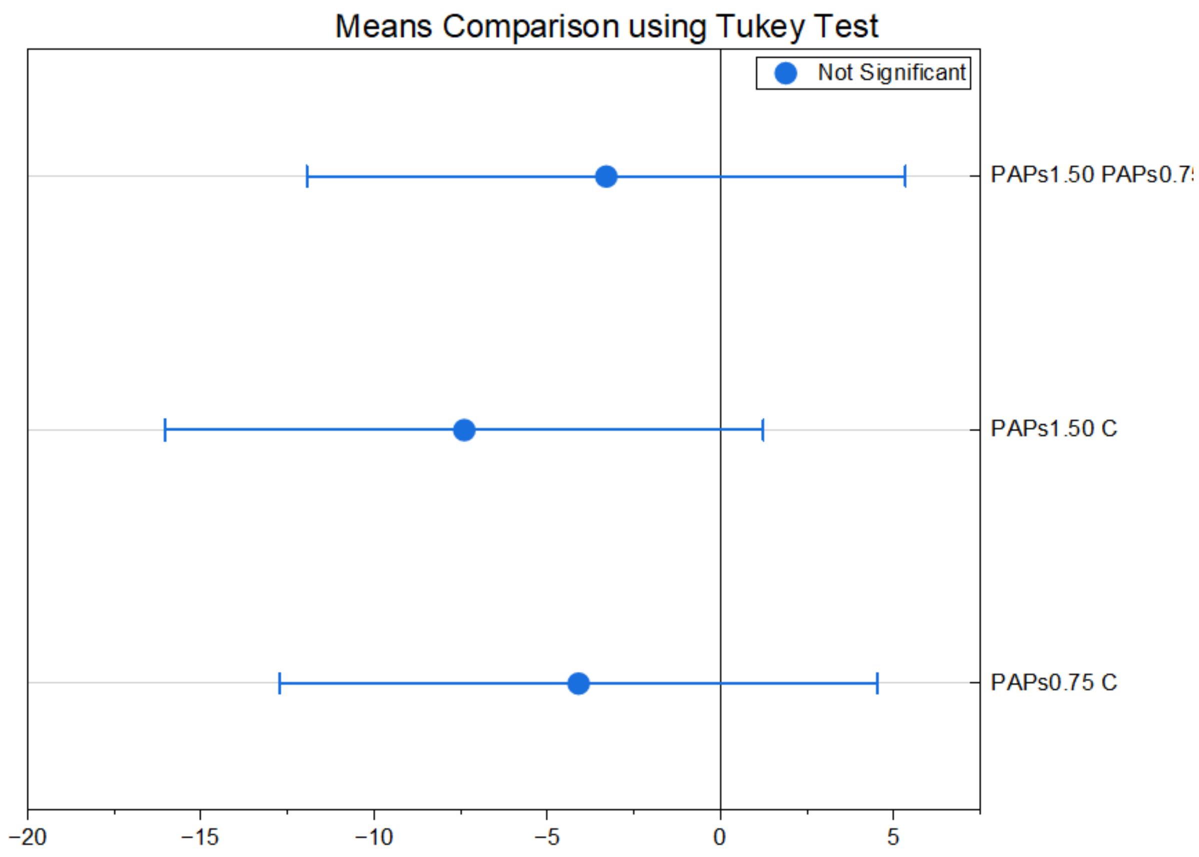

⊟ Power

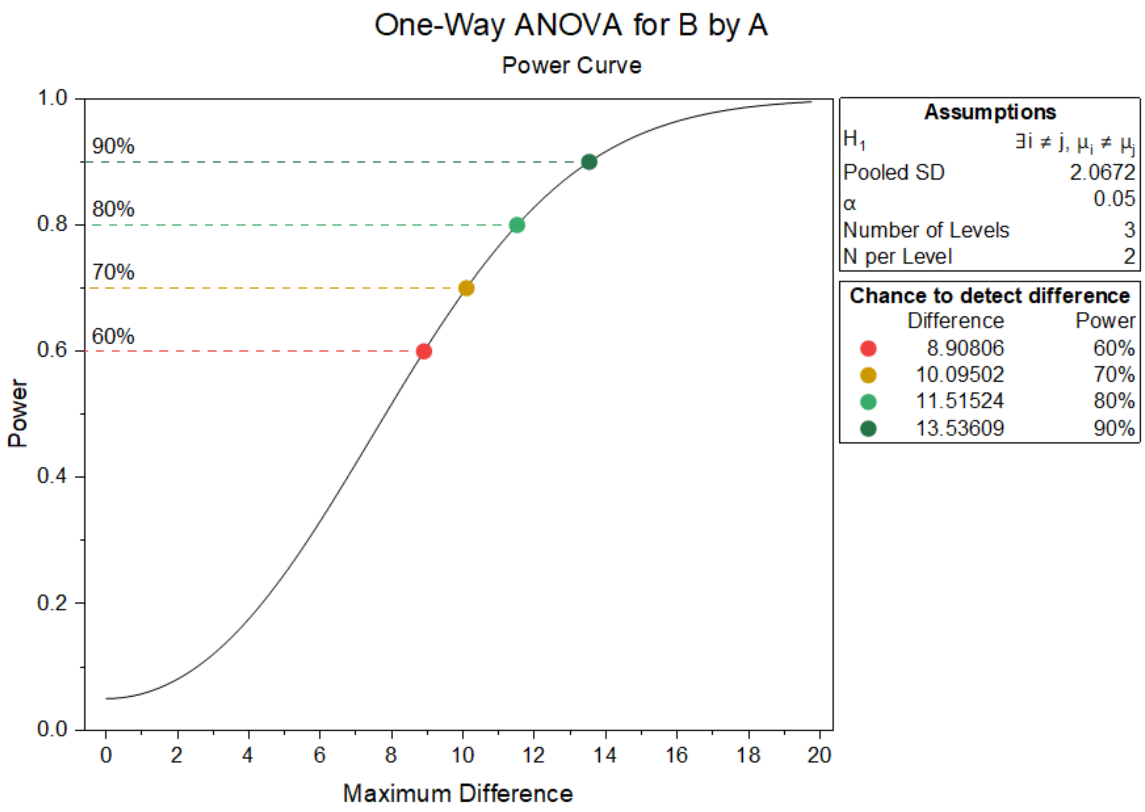

The power curve shows how the statistical power of the test changes as the difference in means varies. Power is the probability of rejecting the null hypothesis when the true difference differs from 0. A power of 80% is commonly considered sufficient. If the power at the expected difference is too low, consider increasing the sample size.

Section S4: ANOVA Results of Hydroponic test for *Lepidium sativum*

Descriptive Statistics

	N Analysis	N Missing	Mean	Standard Deviation	SE of Mean
C	2	0	7.2	0.84853	0.6
PAPs0.75	2	0	6.7	1.41421	1
PAPs1.50	2	0	6.2	1.69706	1.2

ANOVA

Overall ANOVA

	DF	Sum of Squares	Mean Square	F Value	Prob>F
Model	2	1	0.5	0.26786	0.78157
Error	3	5.6	1.86667		
Total	5	6.6			

Null Hypothesis: The means of all levels are equal.
Alternative Hypothesis: The means of one or more levels are different.
At the 0.05 level, the population means are not significantly different.

Fit Statistics

R-Square	Coeff Var	Root MSE	Data Mean
0.15152	0.20392	1.36626	6.7

Means Comparisons

Tukey Test

	MeanDiff	SEM	q Value	Prob	Alpha	Sig	LCL	UCL
PAPs0.75 C	-0.5	1.36626	0.51755	0.93049	0.05	0	-6.20921	5.20921
PAPs1.50 C	-1	1.36626	1.0351	0.76397	0.05	0	-6.70921	4.70921
PAPs1.50 PAPs0.75	-0.5	1.36626	0.51755	0.93049	0.05	0	-6.20921	5.20921

Grouping Letters Table

Tukey Test

	Mean	Groups
C	7.2	A
PAPs0.75	6.7	A
PAPs1.50	6.2	A

Means that do not share a letter are significantly different.

Sig equals 1 indicates that the difference of the means is significant at the 0.05 level.
Sig equals 0 indicates that the difference of the means is not significant at the 0.05 level.

Homogeneity of Variance Test

Levene's Test(Absolute Deviations)

	DF	Sum of Squares	Mean Square	F Value	Prob>F
Model	2	0.37333	0.18667	1.41977E30	<0.0001
Error	3	3.9443E-31	1.31477E-31		

At the 0.05 level, the population variances are significantly different.

Powers

	Alpha	Sample Size	Power
Actual Power	0.05	6	0.06754

The sample size is the total size for all groups.

Summary

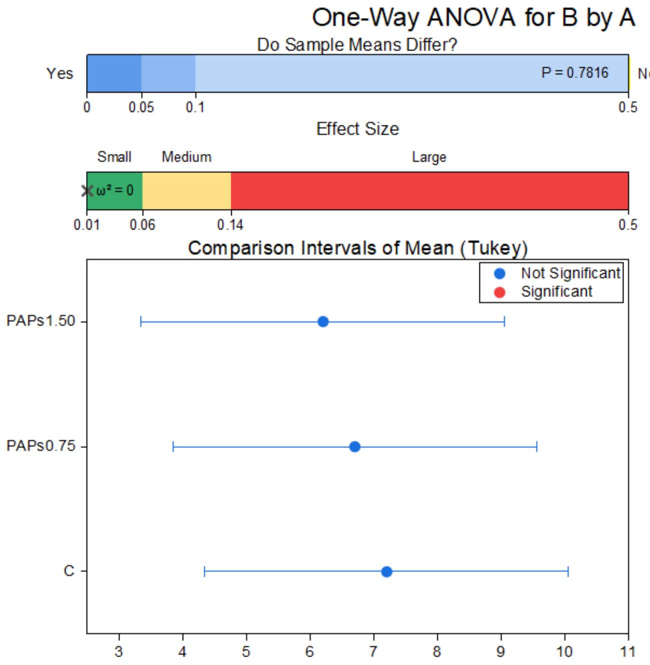

Which means differ?

#	Sample	Differs From
1	PAPs1.50	
2	PAPs0.75	
3	C	

Statistics

Sample	Mean	SD	95% CI of Mean
PAPs1.50	6.2	1.697	(-9.047, 21.447)
PAPs0.75	6.7	1.414	(-6.006, 19.406)
C	7.2	0.849	(-0.424, 14.824)

- At the 0.05 level, the population means are not significantly different.
- Blue intervals indicate no detected differences between the means.

Diagnostic

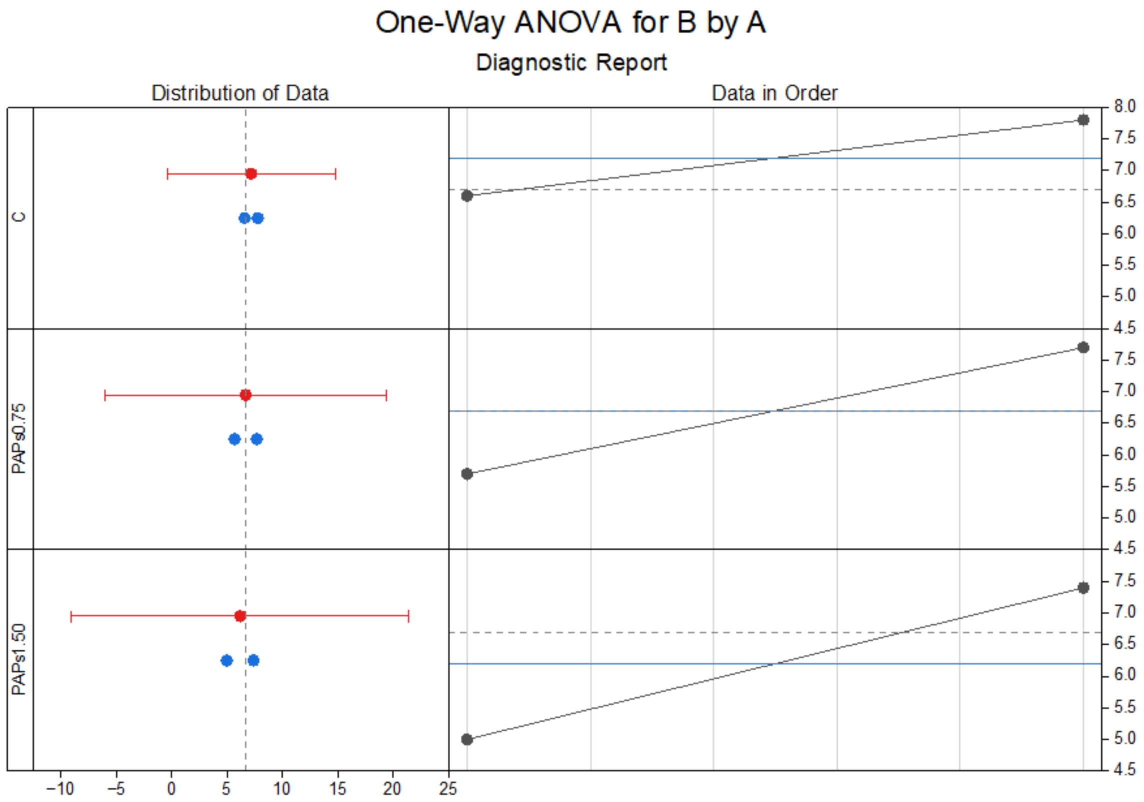

Box Charts

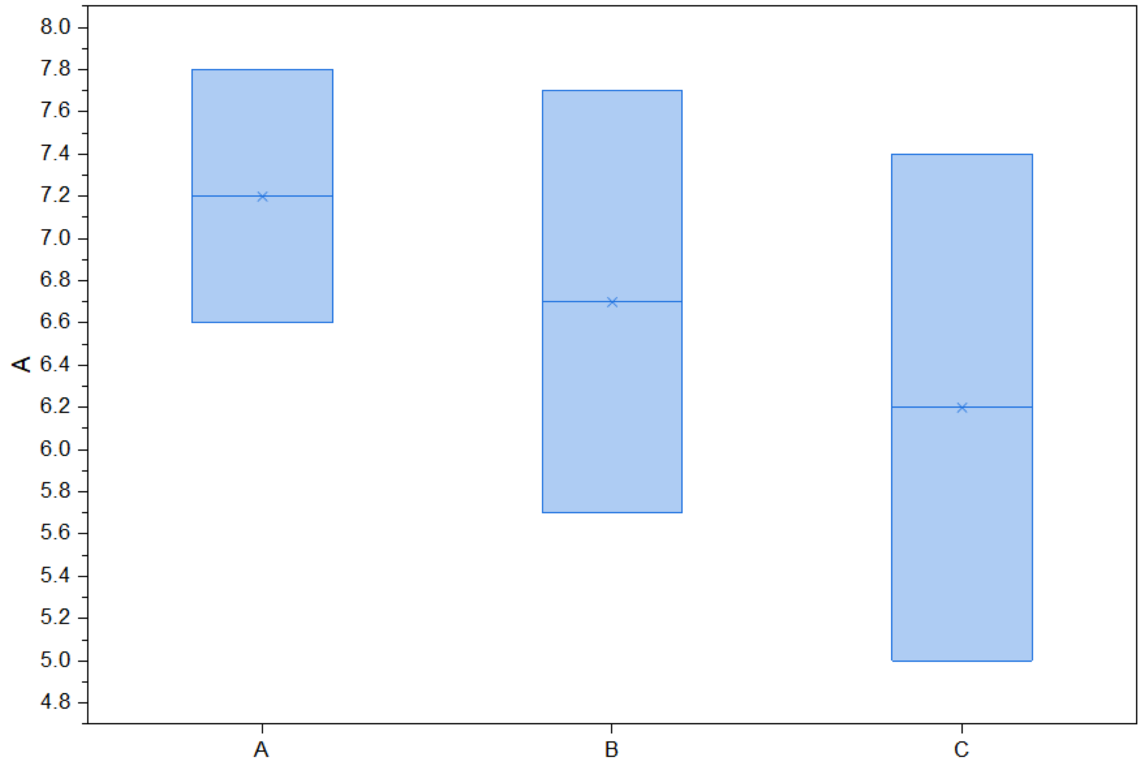

Means Plot (SE as Error)

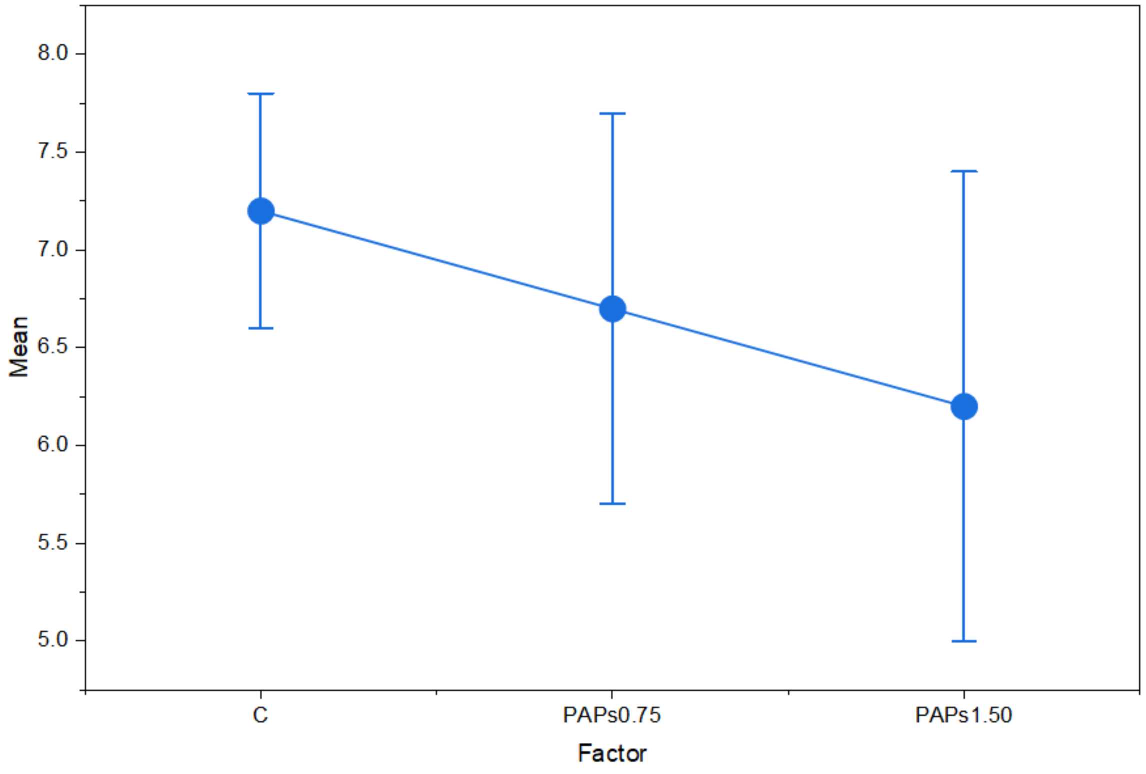

Means Comparison Plot

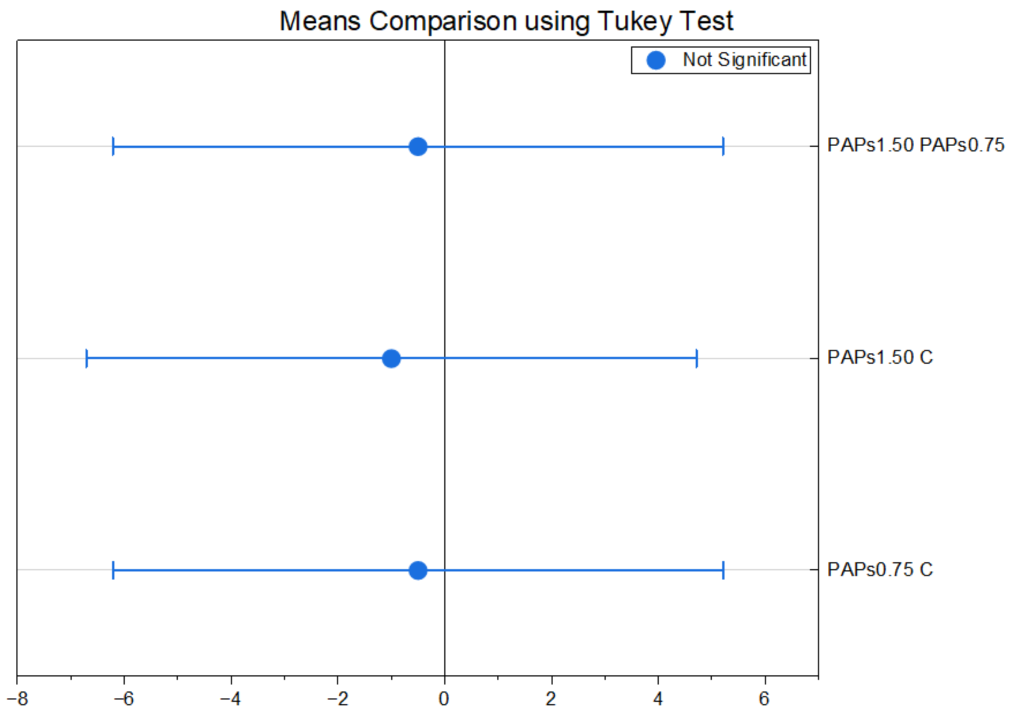

Power

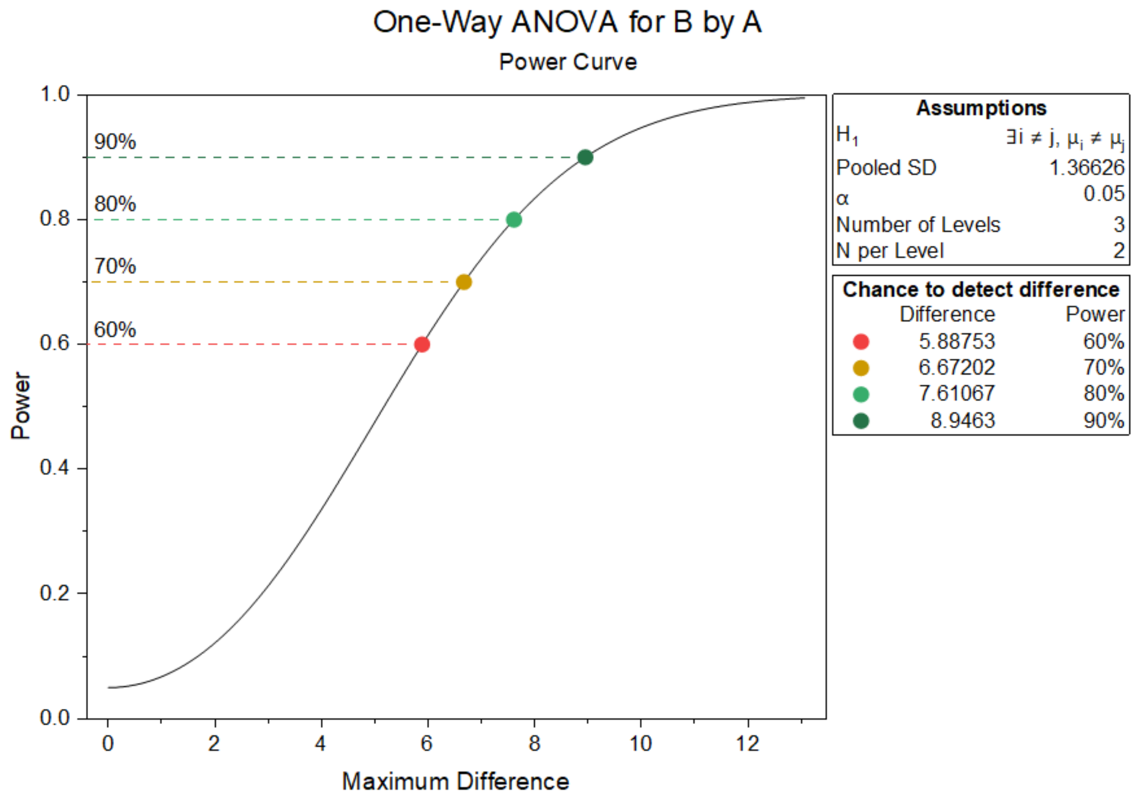

The power curve shows how the statistical power of the test changes as the difference in means varies. Power is the probability of rejecting the null hypothesis when the true difference differs from 0. A power of 80% is commonly considered sufficient. If the power at the expected difference is too low, consider increasing the sample size.

Section S5: ANOVA Results of Pot test for *Lepidium sativum*

Descriptive Statistics

	N Analysis	N Missing	Mean	Standard Deviation	SE of Mean
PAPs1	4	0	7.225	0.95699	0.4785
PAPs5	4	0	7.825	0.49917	0.24958
PAPs10	4	0	5.575	1.18989	0.59494
PAPs20	4	0	3.25	0.26458	0.13229
PAPs30	4	0	2.1	0.66833	0.33417
C	4	0	4.5	0.8165	0.40825

ANOVA

Overall ANOVA

	DF	Sum of Squares	Mean Square	F Value	Prob>F
Model	5	99.78708	19.95742	31.81169	<0.0001
Error	18	11.2925	0.62736		
Total	23	111.07958			

Null Hypothesis: The means of all levels are equal.
Alternative Hypothesis: The means of one or more levels are different.
At the 0.05 level, the population means are significantly different.

Fit Statistics

R-Square	Coeff Var	Root MSE	Data Mean
0.89834	0.15594	0.79206	5.07917

Means Comparisons

Tukey Test

	MeanDiff	SEM	q Value	Prob	Alpha	Sig	LCL	UCL
PAPs5 PAPs1	0.6	0.56007	1.51503	0.88639	0.05	0	-1.1799	2.3799
PAPs10 PAPs1	-1.65	0.56007	4.16634	0.07844	0.05	0	-3.4299	0.1299
PAPs10 PAPs5	-2.25	0.56007	5.68138	0.0089	0.05	1	-4.0299	-0.4701
PAPs20 PAPs1	-3.975	0.56007	10.0371	<0.0001	0.05	1	-5.7549	-2.1951
PAPs20 PAPs5	-4.575	0.56007	11.55214	<0.0001	0.05	1	-6.3549	-2.7951
PAPs20 PAPs10	-2.325	0.56007	5.87076	0.0067	0.05	1	-4.1049	-0.5451
PAPs30 PAPs1	-5.125	0.56007	12.94092	<0.0001	0.05	1	-6.9049	-3.3451
PAPs30 PAPs5	-5.725	0.56007	14.45595	<0.0001	0.05	1	-7.5049	-3.9451
PAPs30 PAPs10	-3.475	0.56007	8.77457	<0.0001	0.05	1	-5.2549	-1.6951
PAPs30 PAPs20	-1.15	0.56007	2.90382	0.35288	0.05	0	-2.9299	0.6299
C PAPs1	-2.725	0.56007	6.88078	0.00148	0.05	1	-4.5049	-0.9451
C PAPs5	-3.325	0.56007	8.39581	1.60952E-4	0.05	1	-5.1049	-1.5451
C PAPs10	-1.075	0.56007	2.71444	0.42258	0.05	0	-2.8549	0.7049
C PAPs20	1.25	0.56007	3.15632	0.27148	0.05	0	-0.5299	3.0299
C PAPs30	2.4	0.56007	6.06014	0.00505	0.05	1	0.6201	4.1799

Grouping Letters Table

Tukey Test

	Mean	Groups				
PAPs5	7.825	A				
PAPs1	7.225	A	B			
PAPs10	5.575		B	C		
C	4.5			C	D	
PAPs20	3.25				D	E
PAPs30	2.1					E

Means that do not share a letter are significantly different.

Sig equals 1 indicates that the difference of the means is significant at the 0.05 level.
Sig equals 0 indicates that the difference of the means is not significant at the 0.05 level.

Homogeneity of Variance Test

Levene's Test(Absolute Deviations)

	DF	Sum of Squares	Mean Square	F Value	Prob>F
Model	5	1.34708	0.26942	1.8833	0.14742
Error	18	2.575	0.14306		

At the 0.05 level, the population variances are not significantly different.

Powers

	Alpha	Sample Size	Power
Actual Power	0.05	24	1

The sample size is the total size for all groups.

Summary

One-Way ANOVA for B by A

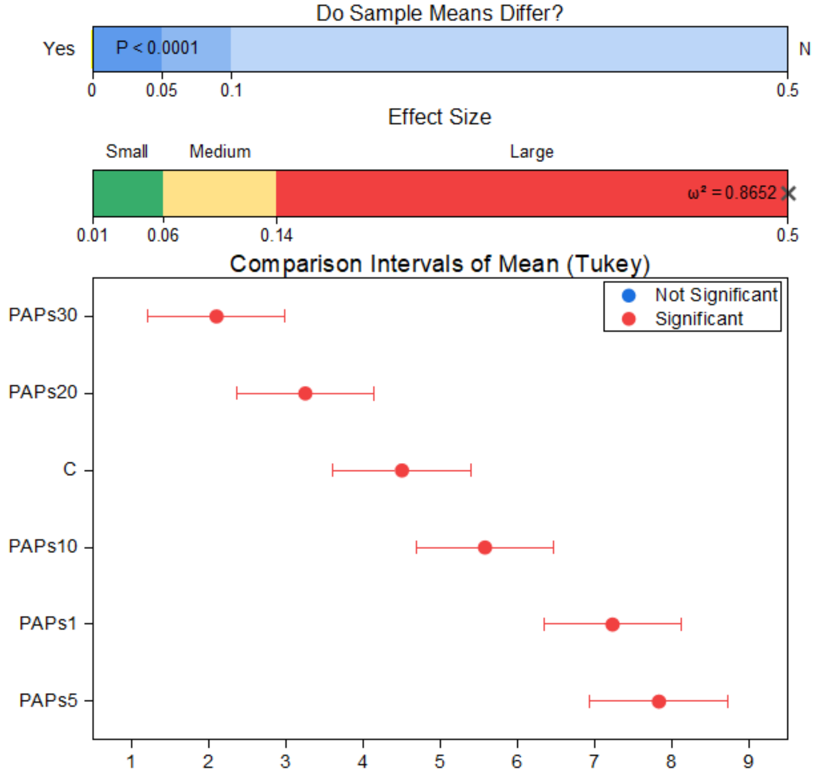

Which means differ?

#	Sample	Differs From
1	PAPs30	3 4 5 6
2	PAPs20	4 5 6
3	C	1 5 6
4	PAPs10	1 2 6
5	PAPs1	1 2 3
6	PAPs5	1 2 3 4

Statistics

Sample	Mean	SD	95% CI of Mean
PAPs30	2.1	0.668	(1.037, 3.163)
PAPs20	3.25	0.265	(2.829, 3.671)
C	4.5	0.816	(3.201, 5.799)
PAPs10	5.575	1.19	(3.682, 7.468)
PAPs1	7.225	0.957	(5.702, 8.748)
PAPs5	7.825	0.499	(7.031, 8.619)

- At the 0.05 level, the population means are significantly different.
- Non-overlapping red intervals indicate differences between the means; consider their size for practical significance.

Diagnostic

One-Way ANOVA for B by A

Diagnostic Report

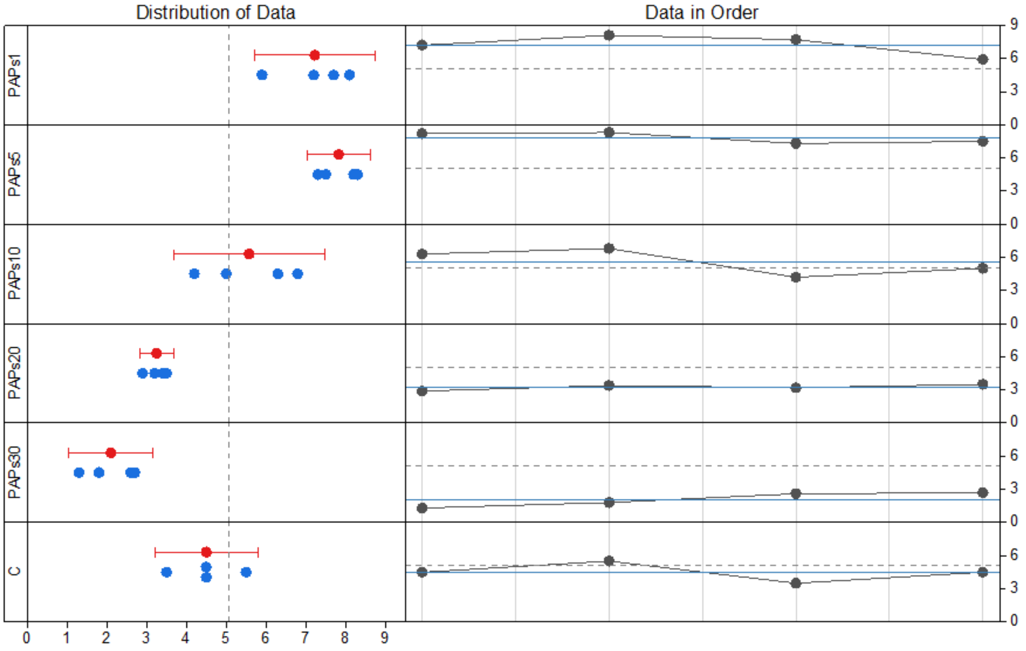

Box Charts

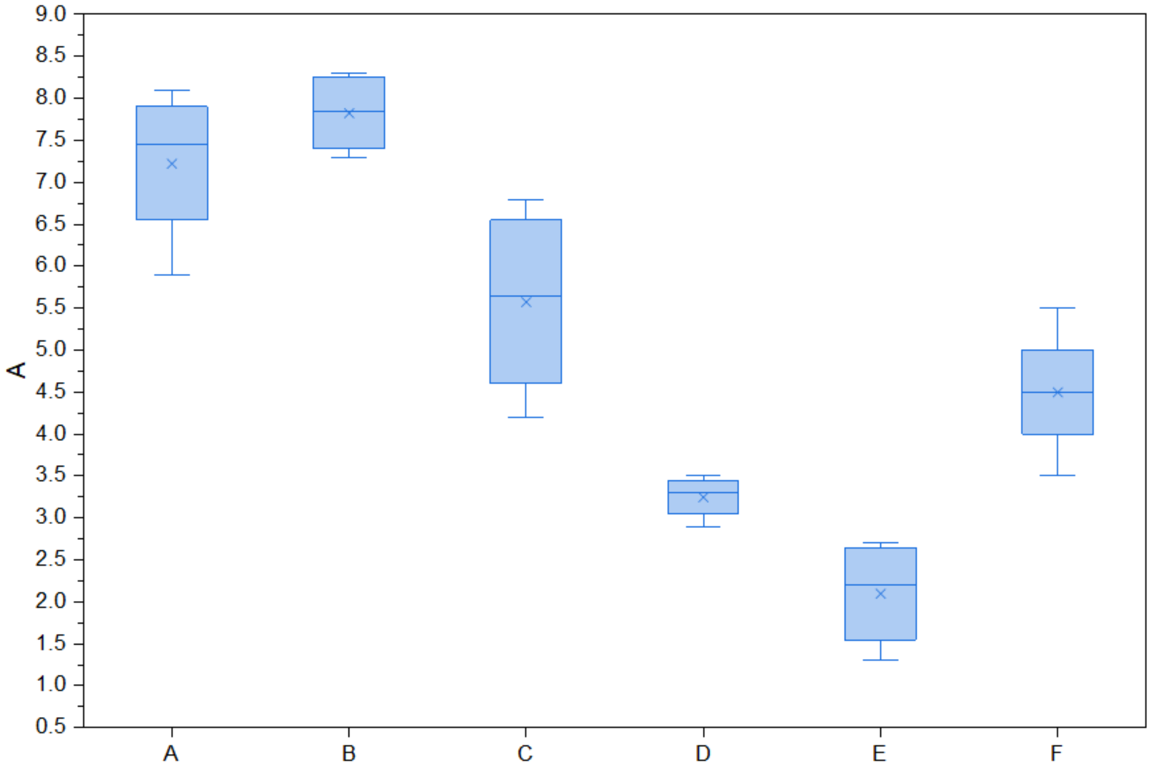

Means Plot (SE as Error)

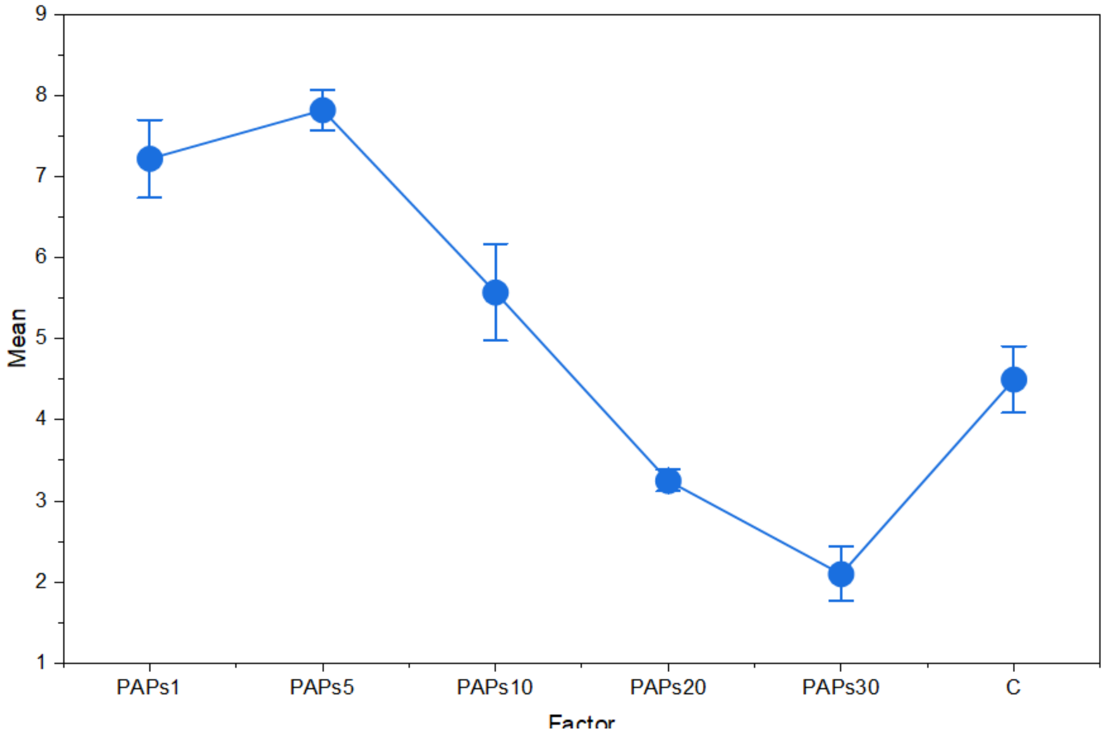

Means Comparison Plot

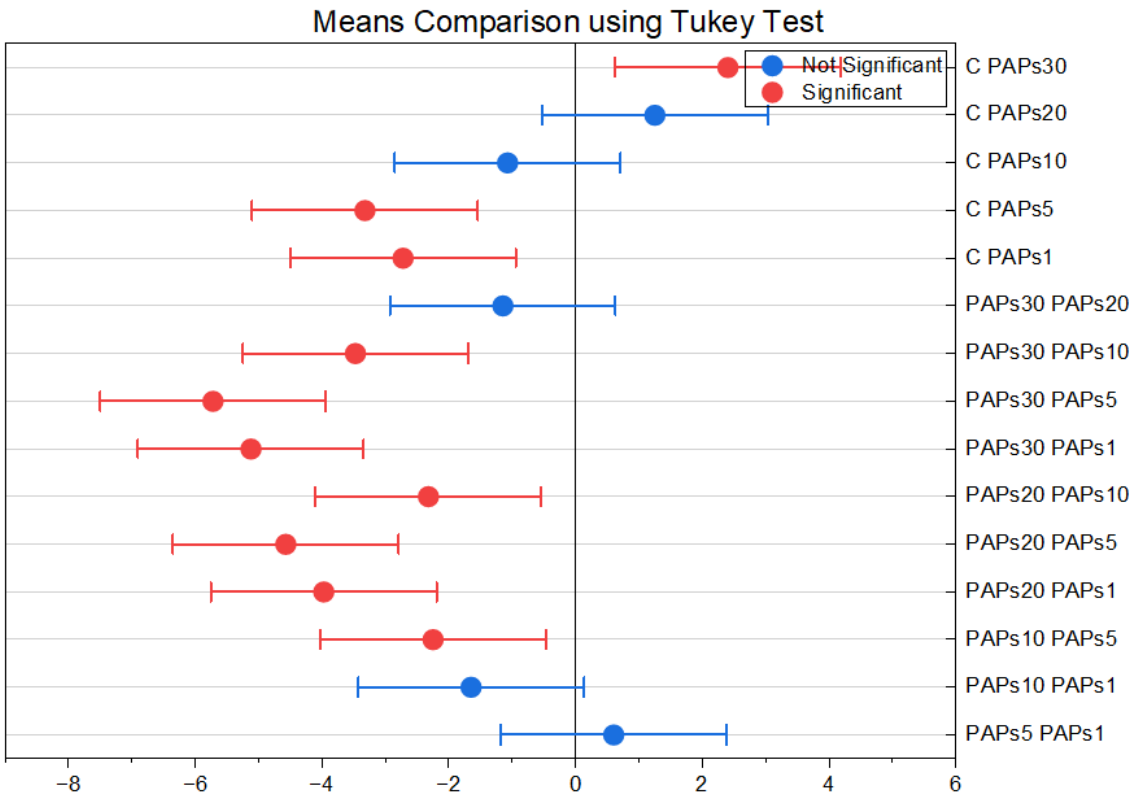

Power

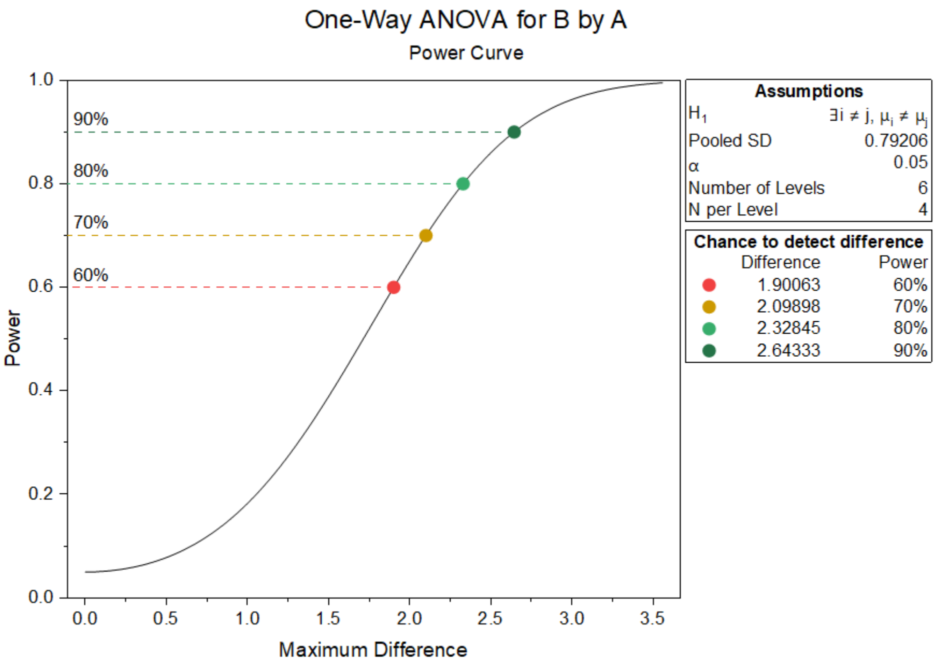

The power curve shows how the statistical power of the test changes as the difference in means varies. Power is the probability of rejecting the null hypothesis when the true difference differs from 0. A power of 80% is commonly considered sufficient. If the power at the expected difference is too low, consider increasing the sample size.

Table S1. Analytical parameters of quantitative analysis (ICP-OES)

Element	Wavelength (nm)	Calibration equation	R^2	LOD sample (mg kg^{-1} d.w.)	LOQ sample (mg kg^{-1} d.w.)
P	213.618	Intensity = 1341 × Concentration − 3.379	0.9999	8.1	26.8
Sn	283.998	Intensity = 1008 × Concentration + 13.06	0.99907	3.3	10.9
Na	589.592	Intensity = 607296 × Concentration + 4398	0.9999	2.5	8.5
S	181.972	Intensity = 321.6 × Concentration + 32.04	0.9999	2.2	7.4
Sb	206.834	Intensity = 455.5 × Concentration − 44.30	0.9999	1.3	4.2
Pb	283.305	Intensity = 2263 × Concentration + 34.62	1.00000	0.91	3.0
Pt	214.424	Intensity = 1604 × Concentration + 6.103	1.00000	0.54	3.0
W	220.449	Intensity = 580.4 × Concentration + 49.80	0.9999	0.86	3.0
Pt	217.468	Intensity = 884.8 × Concentration + 6.701	1.00000	0.89	3.0
Bi	306.771	Intensity = 10234 × Concentration + 12047	0.9999	0.71	2.4
Pb	220.353	Intensity = 2890 × Concentration + 31.26	0.9999	0.66	2.2
In	230.606	Intensity = 2188 × Concentration + 15.51	0.9999	0.57	1.9
V	292.401	Intensity = 39129 × Concentration + 26.21	0.99999	0.48	1.8
W	207.912	Intensity = 1231 × Concentration + 89.72	0.9999	0.50	1.7
Sb	217.582	Intensity = 239.2 × Concentration + 6.202	0.9999	0.50	1.7
Si	251.611	Intensity = 2105 × Concentration + 73.39	0.9999	0.48	1.6
Au	242.794	Intensity = 2096 × Concentration − 21.43	0.9999	0.46	1.5
K	766.491	Intensity = 77680 × Concentration + 761.9	0.9999	0.43	1.4
As	193.696	Intensity = 1111 × Concentration + 7.007	0.99999	0.43	1.4
Ca	396.847	Intensity = 5662829 × Concentration	0.9999	0.32	1.1
Bi	223.061	Intensity = 4402 × Concentration − 180.1	0.9999	0.31	1.0
Se	196.026	Intensity = 1070 × Concentration + 20.96	0.99996	0.24	0.81
Ge	265.117	Intensity = 728.2 × Concentration + 3.697	0.99996	0.25	0.80
Hg	194.164	Intensity = 2263 × Concentration − 3.995	0.9999	0.17	0.65
Mo	202.032	Intensity = 8202 × Concentration − 33.22	0.99999	0.11	0.35
Al	396.152	Intensity = 64091 × Concentration + 66.48	0.99999	0.10	0.34
Ni	231.604	Intensity = 5401 × Concentration + 17.60	0.9999	0.10	0.34
B	249.772	Intensity = 49540 × Concentration + 132.0	1.00000	0.10	0.32
Fe	238.204	Intensity = 39021 × Concentration + 59.51	0.99995	0.08	0.27
Zn	213.857	Intensity = 40696 × Concentration + 19.12	0.9999	0.08	0.27
Ga	417.204	Intensity = 28953 × Concentration + 172.9	0.99998	0.08	0.26
Mg	279.553	Intensity = 426468 × Concentration + 781.5	0.9999	0.08	0.26
Co	238.892	Intensity = 10515 × Concentration + 32.27	0.9999	0.03	0.11
Cu	327.395	Intensity = 50363 × Concentration + 60.41	1.00000	0.03	0.10
Ti	336.122	Intensity = 166223 × Concentration + 29.03	0.9999	0.02	0.06
La	333.749	Intensity = 103139 × Concentration + 21.42	0.99999	0.02	0.05
Ag	328.068	Intensity = 63921 × Concentration − 217.9	0.99998	0.02	0.05
Cr	267.716	Intensity = 50574 × Concentration + 73.46	0.99989	0.01	0.04
Mn	257.61	Intensity = 335189 × Concentration + 139.0	0.9999	0.004	0.01
Cd	214.439	Intensity = 30593 × Concentration + 10.27	0.9999	0.004	0.01
Be	313.042	Intensity = 2507215 × Concentration + 465.1	0.9999	0.010	0.03
Eu	420.504	Intensity = 497366 × Concentration + 8260	0.9999	0.010	0.03
Ce	446.021	Intensity = 50595 × Concentration + 94.97	0.99996	0.010	0.03

Li	670.783	Intensity = 47275712 × Concentration + 18918	0.9999	0.010	0.03
Ba	455.403	Intensity = 3534281 × Concentration + 124.9	0.9999	0.010	0.03
Sr	407.771	Intensity = 10077829 × Concentration + 227.3	1.00000	0.001	0.002
